# Systematically identifying genetic signatures including novel SNP-clusters, nonsense variants, frame-shift INDELs, and long STR expansions that potentially link to unknown phenotypes existing in dog breeds

**DOI:** 10.1186/s12864-023-09390-6

**Published:** 2023-06-05

**Authors:** Zicheng Li, Zuoheng Wang, Zhiyuan Chen, Heidi Voegeli, Judith H. Lichtman, Peter Smith, Ju Liu, Andrew T. DeWan, Josephine Hoh

**Affiliations:** 1 Department of Chronic Disease Epidemiology, School of Public Health, Yale University, New Haven, CT 06510 USA; 2 Department of Biostatistics, School of Public Health, Yale University, New Haven, CT 06510 USA; 3 Department of Comparative Medicine, School of Medicine, Yale University, New Haven, CT 06510 USA; 4 Medical Research Center, The First Affiliated Hospital of Shandong First Medical University & Shandong Provincial Qianfoshan Hospital, 16766 Jingshi Road, Jinan, Shandong 250014 China; 5 Center for Perinatal Pediatric and Environmental Epidemiology, Yale University, New Haven, CT 06510 USA; 6 Department of Ophthalmology and Visual Science, School of Medicine, Yale University, New Haven, CT 06510 USA; 7 Department of Applied Mathematics, Yale University, New Haven, CT 06510 USA

**Keywords:** Whole genome sequencing experiments and analyses, Dogs as model organism, SNP clusters, INDELs, Short Tandem Repeats

## Abstract

**Background:**

In light of previous studies that profiled breed-specific traits or used genome-wide association studies to refine loci associated with characteristic morphological features in dogs, the field has gained tremendous genetic insights for known dog traits observed among breeds. Here we aim to address the question from a reserve perspective: whether there are breed-specific genotypes that may underlie currently unknown phenotypes. This study provides a complete set of breed-specific genetic signatures (BSGS). Several novel BSGS with significant protein-altering effects were highlighted and validated.

**Results:**

Using the next generation whole-genome sequencing technology coupled with unsupervised machine learning for pattern recognitions, we constructed and analyzed a high-resolution sequence map for 76 breeds of 412 dogs. Genomic structures including novel single nucleotide polymorphisms (SNPs), SNP clusters, insertions, deletions (INDELs) and short tandem repeats (STRs) were uncovered mutually exclusively among breeds. We also partially validated some novel nonsense variants by Sanger sequencing with additional dogs.

Four novel nonsense BSGS were found in the Bernese Mountain Dog, Samoyed, Bull Terrier, and Basset Hound, respectively. Four INDELs resulting in either frame-shift or codon disruptions were found in the Norwich Terrier, Airedale Terrier, Chow Chow and Bernese Mountain Dog, respectively. A total of 15 genomic regions containing three types of BSGS (SNP-clusters, INDELs and STRs) were identified in the Akita, Alaskan Malamute, Chow Chow, Field Spaniel, Keeshond, Shetland Sheepdog and Sussex Spaniel, in which Keeshond and Sussex Spaniel each carried one amino-acid changing BSGS in such regions.

**Conclusion:**

Given the strong relationship between human and dog breed-specific traits, this study might be of considerable interest to researchers and all. Novel genetic signatures that can differentiate dog breeds were uncovered. Several functional genetic signatures might indicate potentially breed-specific unknown phenotypic traits or disease predispositions. These results open the door for further investigations. Importantly, the computational tools we developed can be applied to any dog breeds as well as other species. This study will stimulate new thinking, as the results of breed-specific genetic signatures may offer an overarching relevance of the animal models to human health and disease.

**Supplementary Information:**

The online version contains supplementary material available at 10.1186/s12864-023-09390-6.

## Background

Dogs (Canis lupus familiaris) are known as the first species that was fully domesticated by humans about 12,000 ~ 15,000 years ago [1]. Since then, as reliable companions and assistants to their human counterparts, they have gone through an extensive breeding process to produce stable and specific traits that can help them better adapt to a wide variety of different working environments [2, 3]. Strong selective pressure on certain phenotypes has forced most of the breed-defining gene variations to be quickly homogenized within corresponding breeds to consistently produce stably-inherited desired traits throughout generations [4]. Meanwhile, the strict breed recognition process that only dogs whose parents are both from the same breeds will carry on the pedigree, has also facilitated the maintaining of a closed and homogeneous genetic pool of each established modern dog breed [5], resulting in stretches of signature markers specific to certain breeds [6, 7]. This unique breed development process has thus created hundreds of externally distinct yet internally homogenous dog breeds, in terms of both genetics and phenotypes [8].

Understanding the different genetic structures underlying numerous dog phenotypes has long been the key for humans to improve breeding strategies as well as study the connections between dog traits and their counterparts in humans [9, 10]. In fact, dogs and humans often manifest similar behavioral temperaments and are predisposed to particular disorders [9]. Dogs share a living environment with and develop similar immune responses as humans [11]. Furthermore, dogs serve as an ideal animal model to study the disease etiology and treatment development as previously demonstrated during the discovery of genetic mutations in narcolepsy [12], cancer [13, 14], Duchenne muscular dystrophy [15, 16] and inherited retinal dystrophy [17]. Besides chronic as well as age-related diseases, dogs are affected by the same bacteria and viruses that infect human beings, such as *Borrelia burgdorferi*, the causative agent of Lyme disease and SARS-CoV-2, the virus responsible for COVID. As such, especially following the discovery of insulin to treat diabetes, dogs have been commonly used as subjects for preclinical studies for the development of vaccines and therapeutics [18].

All documented studies have demonstrated strong relationships between human and breed-specific traits in dogs. In fact, selecting proper breeds is crucial when using dogs as effective animal models to discover genetic causes of human traits, as different dog breeds are predisposed to different diseases. Although previous dog genetic studies have greatly enhanced our understanding of genetic components associated with body size [19–21], skull shape [22], coat color [23–25], athleticism [26], behaviors [27–31] and diseases, the genetic underpinning that may distinguish dog breeds has yet to be fully explored. All studies were designed to find associated genotypes with given and known phenotypes observed in certain breeds. Our goal is to understand differences across breeds, given advanced sequencing technology. Those breed-specific genotypes will ultimately allow researchers to detect unknown phenotypes.

To do so, we collected, assembled, and analyzed the whole genome sequencing (WGS) data of dogs from 76 breeds. By applying unsupervised machine learning coupled with stepwise optimizations for large volumes of data, we developed a suite of computational algorithms for pattern recognitions at the whole-genome scale. We constructed a high-resolution genetic signature map that characterized the genetic backbones of each dog breed. We also deciphered the common genetic structures shared by multiple breeds to uncover the complex intertwined relationship between them. Besides, we examined the distinctive genetic backbones of each dog breed and established comprehensive information composed of genome-wide SNPs, INDELs and STRs that are exclusively present in specific breeds. Lastly, we selected and validated breed-specific nonsense variants from WGS analyses of additional dogs by Sanger sequencing.

## Results

### Whole genome sequencing results

We assembled the raw WGS data and mapped the genome-wide single nucleotide polymorphisms as well as short insertions and deletions for each dog. After quality filtering, a total of 22,419,814 bi-allelic SNPs and 5,068,857 bi-allelic short INDELs were identified across 412 dogs from 76 breeds. We further identified STRs from the pool of 3,140,027 multi-allelic INDELs, resulting in 1,294,687 candidate STR loci.

We discovered the unique SNP combinations for each breed included in the study at the whole genome scale. Specifically, the genetic signature for a breed was defined that all dogs in the corresponding breed carry the same SNP combinations. The genetic signatures shared between a pair of breeds was defined that all dogs of both breeds carry the same SNP combinations. We also reported our findings on BSGS on the basis of genome-wide genetic signatures. BSGS to a breed are a small set of genetic signatures that are present in all dogs from the corresponding breed but absent in all dogs from other breeds. In other words, BSGS can distinguish dogs of one breed from the others.

We summarized our results on all three types of BSGS (SNPs, INDELs and STRs) in subsequent subsections followed by our findings on genome-wide genetic signatures.

### SNP BSGS reveal unique regional genetic structure characterizing dog breeds

Sixty-eight of the 76 breeds had breed-specific SNPs, consisting of a total of 27,845 SNPs (Data S5). Among them, a total of 139 nonsynonymous breed-specific SNPs were found in 30 breeds (Table 1) with 120 of them located within genes with known functions. Among the 120 variants, 116 were predicted to be missense variants while the remaining 4 were predicted to be nonsense variants. These uniquely owned signatures with potential amino-acid changing effects provide a prominent variant collection for further studies to investigate their impacts on breed-differentiating traits.

**Table 1:** Detailed information of nonsynonymous breed-specific SNPs identified across breeds

**Breed (sample size)**	**Chromosome**	**Gene harboring each variant**	**Position of the amino acid change on protein**	**Amino acid change**	
				**Amino acid in reference breeds**	**Amino acid in the target breed**
Airedale Terrier (*n* = 4)	chr2	IRX6: Iroquois Homeobox 6	148/443	Arginine	Cysteine
	chr11	LOC119874041: Uncharacterized gene	843/1321	Valine	Glycine
	chr11		859/1321	Proline	Threonine
	chr11		888/1321	Leucine	Methionine
	chr17	C17H2orf78: Chromosome 17 homolog 2 open reading frame 78	190/933	Proline	Serine
	chr18	LOC541568: Uncharacterized gene	276/327	Isoleucine	Methionine
	chr18	DUSP8: Dual Specificity Phosphatase 8	8/625	Arginine	Tryptophan
	chr19	NCKAP5: NCK Associated Protein 5	1805/1952	Leucine	Glutamine
	chr33	GAP43: Growth Associated Protein 43	236/243	Arginine	Histidine
Akita (*n* = 5)	chr1	LOC119870150: Uncharacterized gene	156/233	Serine	Phenylalanine
	chr5	ARHGEF12: Rho Guanine Nucleotide Exchange Factor 12	468/1543	Methionine	Isoleucine
	chr11	NFX1: Nuclear Transcription Factor, X-Box Binding 1	667/1118	Leucine	Phenylalanine
	chr11	AQP3: Aquaporin 3	13/324	Glutamic acid	Lysine
	chr11	NOL6: Nucleolar Protein 6	322/1146	Phenylalanine	Leucine
	chr15	LOC106559783: Uncharacterized gene	122/210	Alanine	Valine
	chr16	KLKB1: Kallikrein B1	147/690	Threonine	Arginine
	chr17	EIPR1: EARP Complex And GARP Complex Interacting Protein 1	44/317	Serine	Glycine
	chr20	SLMAP: Sarcolemma Associated Protein	457/865	Serine	Alanine
	chr20	NIBAN3: Niban Apoptosis Regulator 3	559/623	Asparagine	Serine
	chr20		552/623	Glycine	Arginine
	chr20		465/623	Asparagine	Aspartic acid
	chr20	JSRP1: Junctional Sarcoplasmic Reticulum Protein 1	80/391	Leucine	Proline
	chrX	LOC119863881: Uncharacterized gene	107/174	Aspartic acid	Glycine
	chrX	LOC119863881: Uncharacterized gene	113/174	Leucine	Phenylalanine
Alaskan Malamute (*n* = 4)	chr3	SLC2A9: Solute Carrier Family 2 Member 9	426/435	Glycine	Arginine
	chr6	BTBD8: BTB Domain Containing 8	636/1731	Arginine	Lysine
	chr13	TMEM71: Transmembrane Protein 71	59/311	Tyrosine	Histidine
	chr26	LOC119866109: Uncharacterized gene	100/287	Arginine	Glycine
	chr28	PIK3AP1: Phosphoinositide-3-Kinase Adaptor Protein 1	492/821	Asparagine	Serine
	chr33	FBXO40: F-Box Protein 40	565/712	Leucine	Phenylalanine
	chr33	GOLGB1: Golgin B1	2910/2930	Histidine	Tyrosine
Basset Hound (*n* = 6)	chr6	ATP5J2:ATP Synthase Membrane Subunit F	686/752	Tyrosine	Cysteine
	chr6	MYH16: Myosin Heavy Chain 16	1746/1993	Glutamine	Stop
	chr6	KDELR2: Endoplasmic Reticulum Protein Retention Receptor 2	132/207	Arginine	Glycine
Bernese Mountain Dog (*n* = 5)	chr1	MEGF8: Multiple EGF Like Domains 8	2514/2792	Arginine	Glutamine
	chr3	SLC28A1: Solute Carrier Family 28 Member 1	8/629	Arginine	Stop
	chr5	TM4SF5: Transmembrane 4 L Six Family Member 5	77/197	Glycine	Serine
	chr5	ANGPTL3: Angiopoietin Like 3	228/459	Leucine	Phenylalanine
	chr16	FAT1: FAT Atypical Cadherin 1	925/4603	Proline	Arginine
Border Terrier (*n* = 4)	chr7	LOXHD1: Lipoxygenase Homology PLAT Domains 1	518/2224	Asparagine	Serine
Boxer (*n* = 4)	chr9	MYADML2: Myeloid Associated Differentiation Marker Like 2	128/307	Arginine	Glycine
	chr9	SAP30BP: SAP30 Binding Protein	54/322	Threonine	Asparagine
	chr9	MYO15B: Myosin XVB	1577/2964	Histidine	Tyrosine
	chr9	OTOP2: Otopetrin 2	471/590	Serine	Isoleucine
	chr9	CD300A: Cluster of Differentiation 300A	32/330	Serine	Leucine
	chr11	MLLT3: Mixed-Lineage Leukemia Translocated To Chromosome 3 Protein	250/568	Methionine	Threonine
	chr14	GATAD1: GATA Zinc Finger Domain Containing 1	158/325	Methionine	Valine
	chr14	PEX1: Peroxisomal Biogenesis Factor 1	616/1416	Isoleucine	Valine
	chr32	SYNPO2: Synaptopodin 2	1099/1267	Proline	Leucine
Bull Terrier (*n* = 5)	chr1	SELENOV: Selenoprotein V	580/627	Valine	Methionine
	chr1	SIPA1L3: Signal Induced Proliferation Associated 1 Like 3	1409/1803	Threonine	Methionine
	chr9	CACNG5: Calcium Voltage-Gated Channel Auxiliary Subunit Gamma 5	189/275	Threonine	Serine
	chr9	TP53I13: Tumor Protein P53 Inducible Protein 13	91/394	Arginine	Glutamine
	chr20	ADAMTS10: A Disintegrin And Metalloproteinase With Thrombospondin Motifs 10	394/1103	Isoleucine	Threonine
	chr22	PIBF1: Progesterone Immunomodulatory Binding Factor 1	719/723	Lysine	Stop
	chr25	RP1L1: Retinitis Pigmentosa 1-Like 1 Protein	425/1956	Leucine	Methionine
Bullmastiff (*n* = 5)	chr3	LOC100855743: Uncharacterized gene	107/330	Aspartic acid	Glycine
	chr4	LOC111093318: Uncharacterized gene	68/166	Arginine	Histidine
	chr9	WDR81: WD Repeat Domain 81	185/1949	Alanine	Threonine
	chr9	OR3A1H: Olfactory receptor family 3 subfamily A member 1H	232/315	Arginine	Cysteine
	chr9	CACFD1: Calcium Channel Flower Domain Containing 1	165/172	Threonine	Methionine
	chr17	LOXL3: Lysyl oxidase like 3	267/804	Leucine	Phenylalanine
Chow Chow (*n* = 4)	chr2	C2H16orf78: Chromosome 2 homolog 16 open reading frame 78	249/264	Glutamic acid	Aspartic acid
	chr6	ZKSCAN5: Zinc Finger With KRAB And SCAN Domains 5	291/835	Valine	Isoleucine
	chr6	UBN1: Ubinuclein 1	872/1169	Serine	Leucine
	chr6	SLX4: SLX4 Structure-Specific Endonuclease Subunit	95/1733	Glutamic acid	Lysine
	chr6	IFT140: Intraflagellar Transport 140	622/1459	Arginine	Tryptophan
	chr6	CAPN15: Calpain 15	387/1153	Proline	Leucine
	chr8	LTBP2: Latent Transforming Growth Factor Beta Binding Protein 2	674/1817	Threonine	Alanine
	chr12	CEP162: Centrosomal Protein 162	419/1426	Arginine	Glutamine
	chr20	NBEAL2: Neurobeachin-Like Protein 2	2236/2745	Glycine	Aspartic acid
	chr21	LOC119864893: Uncharacterized gene	275/319	Glutamic acid	Aspartic acid
	chr21	OR56A9: olfactory receptor family 56 subfamily A member 9	152/315	Asparagine	Serine
	chr26	MPHOSPH9: M-Phase Phosphoprotein 9	891/1182	Threonine	Isoleucine
	chr32	C32H4orf54: Chromosome 32 homolog 4 open reading frame 54	260/1803	Glycine	Arginine
Collie (*n* = 6)	chr8	CLEC14A: C-Type Lectin Domain Containing 14A	241/489	Glycine	Alanine
	chr18	LOC102157137: Uncharacterized gene	152/310	Tryptophan	Arginine
Dalmatian (*n* = 5)	chr3	SLC2A9: Solute Carrier Family 2 Member 9	188/535	Cysteine	Phenylalanine
	chr10	ANKRD53: Ankyrin Repeat Domain 53	326/507	Serine	Glycine
Doberman Pinscher (*n* = 4)	chr2	PLA2G5: Phospholipase A2 Group V	164/190	Arginine	Tryptophan
	chr5	ACSF3: Acyl-CoA Synthetase Family Member 3	473/600	Arginine	Tryptophan
	chr16	STAR: Steroidogenic acute regulatory protein	67/285	Leucine	Phenylalanine
Field Spaniel (*n* = 4)	chr1	LOC484505: Uncharacterized gene	199/2995	Alanine	Valine
Flat-coated Retriever (*n* = 7)	chr2	LACTBL1: Lactamase Beta Like 1	193/590	Methionine	Isoleucine
Irish Wolfhound (*n* = 4)	chr9	SDK2: Sidekick Cell Adhesion Molecule 2	83/2172	Arginine	Cysteine
	chr14	CRHR2: Corticotropin Releasing Hormone Receptor 2	41/435	Valine	Methionine
	chrX	LOC480918:Uncharacterized gene	77/247	Alanine	Proline
Keeshond (*n* = 5)	chr18	BLVRA: Biliverdin Reductase A	301/353	Valine	Isoleucine
	chr18	PUS7: Pseudouridine Synthase 7	166/659	Phenylalanine	Leucine
Leonberger (*n* = 5)	chr9	GBGT1: Globoside Alpha-1,3-N-Acetylgalactosaminyltransferase 1	303/347	Arginine	Histidine
Manchester Terrier (*n* = 5)	chr6	MYH16: Myosin Heavy Chain 16	329/1993	Glycine	Arginine
	chr6	GPR139: G Protein-Coupled Receptor 139	290/352	Arginine	Lysine
	chr12	RAB44: Member RAS Oncogene Family 44	100/970	Glutamic acid	Lysine
	chr20	DAPK3: Death Associated Protein Kinase 3	383/454	Glutamine	Glutamic acid
	chr21	SERPINH1: Serpin Family H Member 1	14/418	Alanine	Threonine
Miniature Schnauzer (*n* = 5)	chr7	PDC: Phosducin	82/245	Arginine	Glycine
	chr7	MAEL: Maelstrom Spermatogenic Transposon Silencer	85/438	Proline	Alanine
	chr12	GPR63: G Protein-Coupled Receptor 63	324/419	Isoleucine	Threonine
	chr35	LOC488316: Uncharacterized gene	40/487	Arginine	Cysteine
	chr35		95/487	Glutamine	Stop
Newfoundland (*n* = 4)	chr5	ATP2C2: ATPase Secretory Pathway Ca2 + Transporting 2	179/945	Histidine	Leucine
Norwich Terrier (*n* = 4)	chr5	SLC12A4: Solute Carrier Family 12 Member 4	651/1094	Leucine	Valine
	chr5	CTRL: Chymotrypsin-like protease	227/268	Glycine	Arginine
	chr5	PLEKHG4: Pleckstrin Homology And RhoGEF Domain Containing G4	672/1195	Threonine	Serine
	chr18	TPCN2: Two pore segment channel 2	175/926	Valine	Methionine
	chr18	SNX32: Sorting Nexin 32	323/417	Arginine	Tryptophan
	chr18	PCNX3: Pecanex homolog 3	576/2042	Arginine	Tryptophan
	chr31	ZBTB21: Zinc Finger and BTB Domain Containing 21	300/1057	Glycine	Arginine
Rhodesian Ridgeback (*n* = 3)	chr3	SEL1L3: SEL1L family member 3	452/1156	Proline	Leucine
	chr17	TOGARAM2: TOG Array Regulator Of Axonemal Microtubules 2	968/1146	Arginine	Serine
Samoyed (*n* = 5)	chr9	LGALS9: Galectin 9	190/355	Alanine	Proline
	chr9	ERAL1: Era Like 12S Mitochondrial RRNA Chaperone 1	406/437	Arginine	Histidine
	chr20	SH2D3A: SH2 Domain Containing 3A	531/602	Glycine	Serine
	chr38	SLAMF8: SLAM Family Member 8	5/289	Tryptophan	Stop
Scottish Terrier (*n* = 3)	chr2	SREK1: Splicing Regulatory Glutamine/lysine-rich Protein 1	458/647	Arginine	Serine
	chr17	SH2D6: SH2 Domain Containing 6	179/357	Threonine	Proline
St. Bernard (*n* = 6)	chr30	PRTG: Protogenin	553/1200	Arginine	Cysteine
Staffordshire Bull Terrier (*n* = 5)	chr35	H2AC8: H2A Clustered Histone 8	133/133	Tyrosine	Serine
Sussex Spaniel (*n* = 5)	chr2	CYLD: Lysine 63 Deubiquitinase	277/956	Aspartic acid	Asparagine
	chr3	SEL1L3: SEL1L Family Member 3	8/1156	Histidine	Proline
	chr5	COG4: Component Of Oligomeric Golgi Complex 4	682/788	Methionine	Leucine
	chr6	EIF3B: Eukaryotic Translation Initiation Factor 3 Subunit B	735/785	Alanine	Threonine
	chr6	GDPD3: Glycerophosphodiester Phosphodiesterase Domain Containing 3	184/320	Serine	Isoleucine
	chr7	NENF: Neudesin Neurotrophic Factor	108/174	Aspartic acid	Glutamic acid
	chr9	PRRC2B: Proline Rich Coiled-Coil 2B	102/2228	Threonine	Methionine
	chr9	LCN2: Lipocalin 2	202/207	Arginine	Cysteine
	chr11	LOC119873934: Uncharacterized gene	134/142	Leucine	Stop
	chr11	RUSC2: RUN And SH3 Domain Containing 2	1197/1512	Glutamine	Arginine
	chr11	OR13E1: Olfactory Receptor Family 13 Subfamily E Member 1	120/326	Arginine	Histidine
	chr12	COL11A2: Collagen Type XI Alpha 2 Chain	93/1778	Arginine	Proline
	chr15	OTOGL: Otogelin Like protein	1194/2343	Histidine	Glutamine
	chr27	LMBR1L: Limb Development Membrane Protein 1 Like protein	229/489	Valine	Alanine
	chr30	CEP152: Centrosomal Protein 152	849/1718	Valine	Isoleucine
	chrX	HDAC6: Histone Deacetylase 6	130/1217	Glutamine	Arginine
	chrX	CXHXorf66: chromosome X CXorf66 homolog	259/376	Arginine	Cysteine
Tibetan Terrier (*n* = 4)	chr27	LOC119866324: Uncharacterized gene	191/293	Serine	Asparagine
	chr27	NANOGNB: NANOG Neighbor Homeobox	208/328	Proline	Leucine
Welsh Terrier (*n* = 4)	chr11	BNC2: Basonuclin 2	321/1136	Proline	Leucine
	chr27	ABCD2: ATP Binding Cassette Subfamily D Member 2	631/742	Tyrosine	Cysteine

Besides, we identified 18 breeds each carrying at least 10 contiguous breed-specific SNPs in the short genomic regions of about 1 kb in length. They were Airedale Terrier, Akita, Alaskan Malamute, Border Terrier, Cairn Terrier, Chow Chow, Collie, Doberman Pinscher, Field Spaniel, Irish Wolfhound, Keeshond, Manchester Terrier, Miniature Schnauzer, Norwich Terrier, Rhodesian Ridgeback, Rottweiler, Sussex Spaniel, and Tibetan Terrier (Table 2). These signature-enriched short segments each remarked a genomic region that was specifically selected within a certain breed, which were likely to have impact on breed-defining phenotypes.

**Table 2:** High-density (≥ 10 SNP/kb) breed-specific SNP stretch with at least 10 signatures

**Breed**	**Chromosome**	**Spanned contiguous genomic region**	**Fucntional annotation of the enriched region**	**Type(s) of variants within the block**	**Number of unique variants within the enriched region**	**Length of the enriched genomic region (kb)**	**Average density within the enriched region (variants/kb)**
Airedale Terrier	chr17	50532539-50533705	NOTO-RAB11FIP5	intergenic variant	13	1.17	11.15
Akita	chr3	43820580-43822250	LOC119871302-LOC102154570	intergenic variant	17	1.67	10.18
	chr6	67004374-67005252	LOC100685738-LOC111096528	intergenic variant	12	0.88	13.67
	chr11	73476419-73477544	LOC102152863-LOC119874118	intergenic variant	13	1.13	11.56
	chr13	7867964-7869356	ABRA-LOC102153821	intergenic variant	15	1.39	10.78
	chr15	60907864-60908601	MARCHF1	intron variant	11	0.74	14.93
	chr39	2847054-2848043	LOC119863881	upstream gene variant	10	0.99	10.11
	chr39	2851352-2852948	LOC119863881	intron variant missense variant	17	1.60	10.65
Alaskan Malamute	chr2	27213595-27214874	LOC100683304-LOC119870627	intergenic variant	13	1.28	10.16
	chr2	27216309-27219197	LOC100683304-LOC119870627	intergenic variant	29	2.89	10.04
	chr24	43606138-43607724	LOC119865702-LOC106557684	intergenic variant	16	1.59	10.09
Border Terrier	chr2	61435032-61436207	LOC102154600-IRX5	intergenic variant	12	1.18	10.21
	chr11	72265410-72266330	ASTN2-TLR4	intergenic variant	10	0.92	10.87
	chr33	11685807-11686737	CBLB	intron variant	10	0.93	10.75
Cairn Terrier	chr20	37745278-37746171	DNAH1	intron variant	11	0.89	12.32
Chow Chow	chr2	66264809-66265603	ZNF423	intron variant	11	0.79	13.85
	chr6	73743761-73744749	LOC111096452	upstream gene variant	10	0.99	10.12
	chr16	52339968-52341044	LOC119874732-LOC119874733	intergenic variant	11	1.08	10.22
Collie	chr9	27078819-27080678	TMEM92-XYLT2	intergenic variant	21	1.86	11.30
Doberman Pinscher	chr16	32621842-32622762	LOC111090167-LOC111090291	intergenic variant	11	0.92	11.96
Field Spaniel	chr6	58451107-58452596	LOC106558910	intragenic variant	15	1.49	10.07
Irish Wolfhound	chr28	37068313-37069058	LOC119866537	upstream gene variant	10	0.75	13.42
Keeshond	chr18	15227503-15228450	PUS7	intron variant	10	0.95	10.56
Manchester Terrier	chr14	27287580-27288099	SCIN	intron variant	10	0.52	19.27
Miniature Schnauzer	chr3	65005786-65006868	CD38	downstream gene variant	11	1.08	10.17
	chr12	52151851-52152658	LOC119881688-LOC111098176	intergenic variant	10	0.81	12.39
Norwich Terrier	chr17	18008212-18009270	LOC119869824-LOC100685329	intergenic variant	11	1.06	10.40
Rhodesian Ridgeback	chr3	86344183-86344850	CCDC149	intron variant	10	0.67	14.99
Rottweiler	chr37	27206943-27207602	LOC119867906-LOC119867974	intergenic variant	11	0.66	16.69
Sussex Spaniel	chr7	63646205-63647049	LOC119876414-LOC119872700	intergenic variant	13	0.84	15.40
	chr17	10252987-10255589	LOC610196-LOC106559867	intergenic variant	27	2.60	10.38
	chr17	10256599-10257637	LOC610196-LOC106559867	intergenic variant	12	1.04	11.56
	chr17	10258238-10259968	LOC610196-LOC106559867	intergenic variant	18	1.73	10.40
	chr30	15053870-15055423	LOC111093384-CEP152	intergenic variant	16	1.55	10.30
	chr38	3219161-3220318	KCNT2	intron variant	14	1.16	12.10
Tibetan Terrier	chr3	46951394-46952368	LOC608613-LOC119871198	intergenic variant	10	0.97	10.27

Eight breeds did not have any breed-specific SNP signatures. They were Australian Shepherd, Beagle, Bichon Frise, Dachshund, English Springer Spaniel, Golden Retriever, Labrador Retriever and Pointer. Noteworthily these eight breeds had few genetic signatures compared to other breeds in the collection as depicted above. This could essentially limit the size of candidate signature pool that can uniquely characterize them.

Most breed-specific SNPs were in non-coding regions (98.93%, Table S1). 299 (1.07%) breed-specific SNPs across 30 breeds were in the exons of the protein coding regions. In addition, the number of breed-specific SNPs also demonstrated an uneven distribution across breeds. Four breeds including Sussex Spaniel, Akita, Chow Chow and Alaskan Malamute were found to have the highest number of breed-specific SNPs in both coding and non-coding regions (accounted for 43.75% of all in total). The four breeds also had the highest number of breed-specific SNPs located in the exons. Specifically, Chow Chow had 36 of them, Akita 35, Sussex Spaniel 30, and Alaskan Malamute 19. The relatively high number of breed-specific SNPs of these four breeds partially reflected their overall genetic deviance from all other dog breeds.

We aggregated breed-specific SNPs by their genomic locations and identified 1,226 genomic regions (partitioned by genes) as “signature hot spots” each having at least 5 closely located breed-specific SNPs in the specific breed. These hot spots harbored a total of 15,264 breed-specific SNPs (54.82%), which suggested that these breed-characterizing signatures were enriched in certain gene regions rather than sporadically distributed across the genome. We found two genes that harbored the highest numbers of breed-specific SNPs in two respective breeds. The NKAIN3 gene (sodium/potassium transporting ATPase interacting 3) with 102 breed-specific SNPs was exclusive to Bull Terriers. The STK12 gene (Syntaxin 12) with 76 signatures was exclusive to Flat-coated Retrievers. Additionally, this 31 kb-long segment was also the gene region with the densest breed-specific SNPs distribution (2.49 signatures /kb in average) found across the genome (Table S2).

We exhaustively searched the genome for SNP signatures that were specific to any breed-pairs within the collection. A total of 437 signatures were discovered between 64 different breed-pairs (Table S2). Segments of exclusively shared SNP signatures were observed between all six breed-pairs from the four East Asian breeds as well as the following breed-pairs: Bernese Mountain Dog + Bull Terrier, Bull Terrier + Manchester Terrier, Bull Terrier + Miniature Schnauzer, and Collie + Shetland Sheepdog. The exclusivity of shared signatures stretches in breed pairs provided strong evidence for the common evolution history between different dog breeds before their evolutionary divergence.

### INDEL BSGS identify severe protein-changing variants exclusively existing in a single breed

We extensively scanned the genome for INDELs exclusively presented in a single breed. A total of four coding breed-specific INDELs were identified while one of them was predicted to cause a codon deletion in Bernese Mountain Dog and three of them were predicted to cause frame-shift in the Airedale Terrier, Chow Chow and Norwich Terrier, respectively (Table 3). These four breed-specific coding INDELs provided specific gene targets for further investigation on their predicted high biological impacts.

**Table 3:** Breed-specific INDELs within coding regions

**Breed**	**Chromosome**	**Position**	**Genomic region**	**Variant type**	**Reference allele**	**Alternative allele**	**Position of the first impacted amino acid on protein**
NorwichTerrier	chr5	82436555	ZDHHC1	frameshift variant	CG	C	156/511
Chow Chow	chr8	45457109	SIPA1L1	disruptive inframe deletion	AGTC	A	1641/1806
Bernese Moutain Dog	chr16	48047125	CENPU	conservative inframe deletion	TGAA	T	84/422
Airedale Terrier	chr18	40255586	OR5J2	frameshift variant	AG	A	297/312

A total of 4,341 breed-specific short INDELs (insertion or deletion size less than 10 nucleotides) were identified across 58 breeds (Data S7), which were all covered by the 68 breeds discovered with breed-specific SNPs. The Akita (N_Breed-specific INDELs_ = 543), Sussex Spaniel (N_Breed-specific INDELs_ = 557), Chow Chow (N_Breed-specific INDELs_ = 502) and Alaskan Malamute (N_Breed-specific INDELs_ = 382) were found to have the highest amount of breed-specific INDELs. These four dog breeds also ranked highest with the most breed-specific SNPs, marking their the genetic uniqueness. When evaluated by variant categories, the vast majority of breed-specific INDELs were located within the non-coding region (99.91%), which is significantly higher than 98.93% of the non-coding breed-specific SNPs found (*p*_*Fisher’s exact*_ = 3.16 × 10^–14^). Notably, this is also significantly higher than 99.59% of overall non-coding INDELs found within the dataset (*p*_*Fisher’s exact*_ = 1.88 × 10^–4^). These indicated the importance of breed-specific INDELs in coding regions, as they were mostly nonsynonymous and had a huge impact on the corresponding protein sequences.

### STR BSGS uncovers highly differentiated loci indicating unique mutation history of specific breeds

We further scanned for genome-wide STR signatures that can set a single breed apart from the others regarding the number of repetitive units at each locus. Among the 54 breeds identified with breed-specific STRs, the Akita (N_Breed-specific STRs_ = 109), Alaskan Malamute (N_Breed-specific STRs_ = 54), Chow Chow (N_Breed-specific STRs_ = 64) and Sussex Spaniel (N_Breed-specific STRs_ = 65) again stayed on top of the signature count list, owing to their genetic uniqueness within the 76-breed collection. Forty-two of 604 signatures were found to feature differences of at least three repetitive units between target breeds and reference breeds (Table 4).

**Table 4:** Breed-specific STR expansion/contraction signatures with at least 3 unit of differences

**Breed**	**Chromosome**	**Position**	**Genomic region**	**Variant type**	**Signature type**	**Non-signature allele**	**Signature alleles**
Akita	chr1	54147922	PDE10A	intron variant	Expansion	A(G)0	A(G)3-4
Akita	chr4	59092147	NDST1	intron variant	Contraction	G(AC)3-4	G(AC)0
Akita	chr8	35116671	RTN1	intron variant	Expansion	G(GA)0-1	G(GA)4
Akita	chr11	22063363	ZCCHC10	intron variant	Expansion	C(T)0-1	C(T)4-5
Akita	chr11	22070815	ZCCHC10	upstream gene variant	Expansion	G(CT)0	G(CT)3-4
Akita	chr13	7862683	ABRA	upstream gene variant	Expansion	A(AC)0-3	A(AC)6-8
Akita	chr14	34795865	LOC102155842	intron variant	Contraction	G(TTTA)5-8	G(TTTA)0
Alaskan Malamute	chr4	63385968	LOC111095611-LOC119871717	intergenic region	Expansion	T(A)0-1	T(A)4
Alaskan Malamute	chr5	44129368	SGIP1	intron variant	Expansion	A(TTCT)0-3	A(TTCT)8-10
Alaskan Malamute	chr11	57850532	LOC100684552-LOC102153773	intergenic region	Expansion	T(TG)0-3	T(TG)6-8
Alaskan Malamute	chr16	19525576	DPP6	intron variant	Expansion	C(T)0-2	C(T)6-8
Alaskan Malamute	chr20	30027711	LOC102151998	intron variant	Contraction	A(AC)4-7	A(AC)0
Alaskan Malamute	chr23	27717171	EAF1	upstream gene variant	Expansion	C(T)0-3	C(T)10-12
Alaskan Malamute	chr27	2582967	KRT5-KRT6A	intergenic region	Expansion	T(TG)0-5	T(TG)10-11
Bernese Mountain Dog	chr23	52282322	LOC119865448	intron variant	Contraction	C(T)3-6	C(T)0
Boxer	chr13	62408506	LOC102152779-LOC111098639	intergenic region	Contraction	C(GT)4-8	C(GT)0-1
Bull Terrier	chr13	53005404	LOC119865325-ADGRL3	intergenic region	Expansion	T(G)0-1	T(G)4-5
Bull Terrier	chr17	8952549	LOC111090497-LOC106559885	intergenic region	Expansion	T(TG)0-3	T(TG)6-8
Bull Terrier	chr20	38871990	DOCK3	intron variant	Contraction	A(AG)6-11	A(AG)0
Chinese Shar-pei	chr29	39193715	CDH17-GEM	intergenic region	Contraction	T(G)7-8	T(G)0
Chow Chow	chr8	48904017	TTLL5	intron variant	Expansion	A(T)0	A(T)3-4
Chow Chow	chr14	55772630	MET-LOC119867730	intergenic region	Contraction	A(T)7	A(T)0-3
Chow Chow	chr16	52334579	LOC119874732-LOC119874733	intergenic region	Expansion	T(A)0-1	T(A)5
Collie	chr12	19367375	LOC119874254-LOC119876904	intergenic region	Expansion	C(T)0-3	C(T)8-10
Collie	chr18	44133652	PHF21A	intron variant	Expansion	C(T)0-2	C(T)5-6
Doberman Pinscher	chr13	12142587	LOC102156107	intron variant	Expansion	C(TG)0-2	C(TG)5-6
English Bulldog	chr1	48446198	LOC111095873	intron variant	Expansion	G(AGAT)0-1	G(AGAT)8-11
English Cocker Spaniel	chr4	55189185	LOC119871642-LOC111095573	intergenic region	Contraction	C(A)4-8	C(A)0
English Setter	chr3	73077170	RHOH-N4BP2	intergenic region	Expansion	T(A)0	T(A)4-5
Field Spaniel	chr25	20337021	SH3RF1	intron variant	Contraction	T(A)3-4	T(A)0
Keeshond	chr18	14955131	ATXN7L1	intron variant	Contraction	C(GT)3-4	C(GT)0
Manchester Terrier	chr8	60703113	FOXN3	intron variant	Expansion	T(A)0-2	T(A)5-10
Miniature Pinscher	chr17	46146498	LOC100683097-LRRTM4	intergenic region	Contraction	C(CT)5-6	C(CT)0
Miniature Schnauzer	chr35	25561153	LOC488316	intron variant	Expansion	T(A)0-1	T(A)4
Samoyed	chr19	43429534	LOC111091178-LOC111091179	intergenic region	Expansion	T(TG)0-2	T(TG)5-9
Soft Coated Wheaten Terrier	chr9	6703622	LOC111097523	downstream gene variant	Expansion	C(T)0	C(T)3-5
Staffordshire Bull Terrier	chr3	82791292	LOC119871405-LOC111095398	intergenic region	Expansion	T(A)0-3	T(A)6-7
Sussex Spaniel	chr17	10256981	LOC610196-LOC106559867	intergenic region	Expansion	A(CT)0-3	A(CT)6-7
Sussex Spaniel	chr18	30624250	LOC111091020-LOC102156018	intergenic region	Expansion	T(A)0-2	T(A)6-7
Sussex Spaniel	chr20	16464862	LOC119864813-LOC111091431	intergenic region	Expansion	T(TTG)0-1	T(TTG)5
Sussex Spaniel	chr31	9390314	LOC111093521-LOC119867121	intergenic region	Expansion	C(T)0	C(T)4-6
West Highland White Terrier	chr24	21533963	LOC100687382	intragenic variant	Contraction	C(CT)5-8	C(CT)0-1

From all 1,294,687 candidate STR loci (repetitive unit length between 1–6 bp) identified from the genome, we found 604 breed-specific STR signatures that featured either a large or a small number of repeats in certain breeds compared to all other breeds (Data S8).

One of the variant loci with a large number of copy differences between the high-repeat group and low-repeat group was identified to be within one intron of the SGIP gene (Chr5: 44129368). At this locus, the Alaskan Malamute was found to carry alleles with at least 8 consecutive (TTCT) repetitive units while all other breeds carried less than 3 units at this locus. Three Akita-specific STR signatures (Chr11: 22049322, 22063363, 22070815) were identified in the upstream, intron and downstream regions of the ZCCHC10 gene. All reference breeds only had one repeat or did not have any repeats of three loci while the Akita carried a considerably higher number of repeats. Notably, a long stretch of 51 Akita-specific SNP signatures have also been identified within the gene region of ZCCHC10, which further indicates the genetic uniqueness of the Akita at this specific locus. Furthermore, we found that English Bulldog uniquely carried at least eight units of (AGAT) tetra-nucleotide repeat within LOC111095873 (Chr1: 48446198), contrasting to one or no repeat in the remaining dog breeds. Though the biological function of the corresponding protein had not been yet characterized, the long segment of repeats provided evidence of unique past mutation events that specifically took place and got selected during the breed formation process of the English bulldog.

### SNP-based genetic signatures reveal shared genetic structures and relationships among dog breeds

Four breeds with an East Asian origin including the Chinese Shar-Pei, Chow Chow, Alaskan Malamute and Akita were found to have the largest number of genetic signatures across the genome (Fig S1). The Beagle, Golden Retriever and Dachshund had the lowest number of genetic signatures, both by themselves and shared with other breeds. Of the three, the Dachshund had the lowest number of genetic signatures. We also confirmed that the number of genetic signatures for each breed did not correlate with the sample size of the breed (Pearson-correlation = -0.0926). The complete map and the full genetic signature results are available in Supplementary Materials (Data S3). We present the total numbers of SNP-based GS in Table S6.

**Fig. 1:**
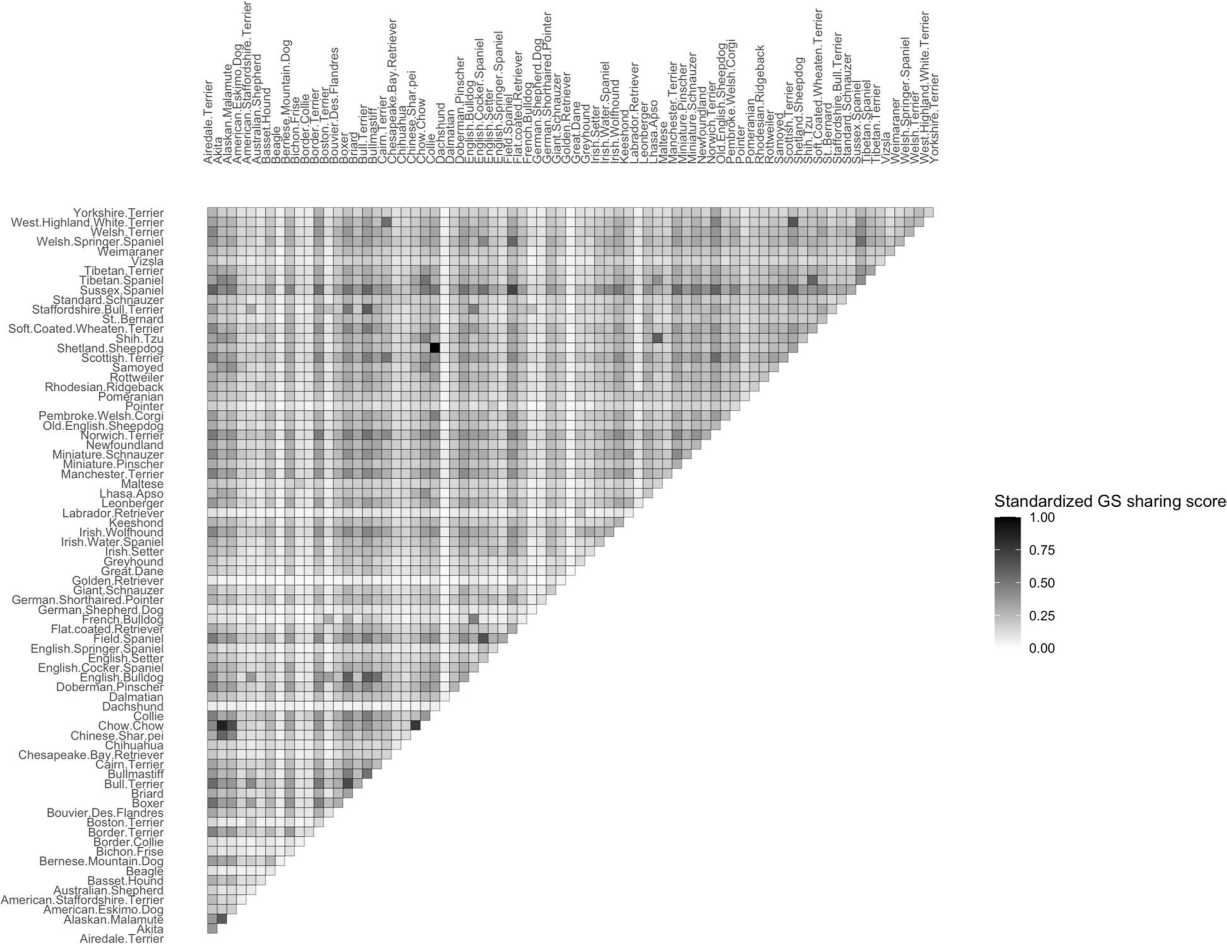
SNP Genetic signature sharing based relatedness between 76 dog breeds

We further investigated the shared genetic signatures between different breeds. By overlapping the genetic signatures of both breeds within each breed pair, we generated a genetic-signature-based relatedness profile for all breed pairs in the 76-breed collection (Fig. 1). Among all breed-pairs, we found that the Collie and Shetland Sheepdog shared the highest proportion of genetic signatures. The shared genetic signatures took up 34.46% of the total genetic signatures discovered in these two breeds (Tables S7 and S8). High proportion of similarity sharing was also observed in morphologically similar breed pairs such as the Akita-Chow-Chow pair (28.80%), Lhasa-Apso-Shih-Tzu pair (23.65%), Field-Spaniel-Sussex-Spaniel pair (25.79%) and Boxer-English-Bulldog pair (24.35%). Low fraction of GS sharing was primarily observed within breed pairs involving the Labrador Retriever, Golden Retriever and Dachshund. The highest proportion of genetic signature sharing was observed to be no more than 10% for all breed pairs involving any of these three breeds. The relatively low number of genetic signatures and high genetic diversity within all these three breeds contributed to this phenomenon. Surprisingly, we found that the English Bulldog was genetically distant to the Chinese Shar-Pei based on the genetic signature sharing score (10.52%) though they had many morphological similarities with each other. This further suggested the existence of considerable unobserved breed-defining traits underlying widely acknowledged morphological traits of dog breeds.

This figure shows the degree of relatedness among 76 breeds. The 76 dog breeds in GS-76 are labeled in alphabetical order on the left and the top. In between, we see 2850 breed pairs. The color of each grid represents relatedness of each corresponding pair of breeds. The color gradient from black to white represents the degree of relatedness. The more related between a breed-pair, the darker the cell would appear.

The above similarity scores were calculated based on SNP GS. To assess the robustness of the breed similarity scores based on the type of variants, we also calculated the similarity scores based on INDEL GS and STR GS discovered across the genome (See Data S10 for detailed similarity score list of each breed) and calculated Spearman’s correlation coefficients (*corr*_Spearman_). Both scores based on INDEL and STR GS showed strong correlations with the scores based on SNP GS for all breed pairs (*corr*_Spearman_ ranged from 0.88 to 0.98 between SNP and INDEL GS, *corr*_Spearman_ ranged from 0.83 to 0.97 between SNP and STR GS) (Table S14).

High correlations across different types of GS showed robustness of the similarity score metric. Three types of variants, SNPs, INDELs and STRs, had different biological aspects, including allele variability, mutation rate and mechanism. The Spearman’s correlation coefficients between STR and SNP GS based similarity scores were lower than the ones between INDEL and SNP GS based similarity scores. It was known that STRs had higher mutation rates compared to SNPs and INDEL. This reflected in the results, for example, based on the SNP GS scores five breeds that were most similar to the French Bulldog were the English Bulldog, Boxer, Bull Terrier, Staffordshire Bull Terrier and Boston Terrier. On the other hand, based on the STR GS scores, English Bulldogs, Boxer and Bull Terrier stayed as the top three to be the most similar breeds with French Bulldog, whereas Staffordshire Bull Terrier and Boston Terrier dropped to the12th and the 25^th^ similar to the French Bulldogs, respectively (Table S15). Considering the French Bulldogs, Staffordshire Bull Terrier and Boston Terrier were established around the same era (Table S10), such a drop of similarity based in STR GS might suggest cross-breeding events with other breeds.

### Genomic structures of BSGS

**Fig. 2:**
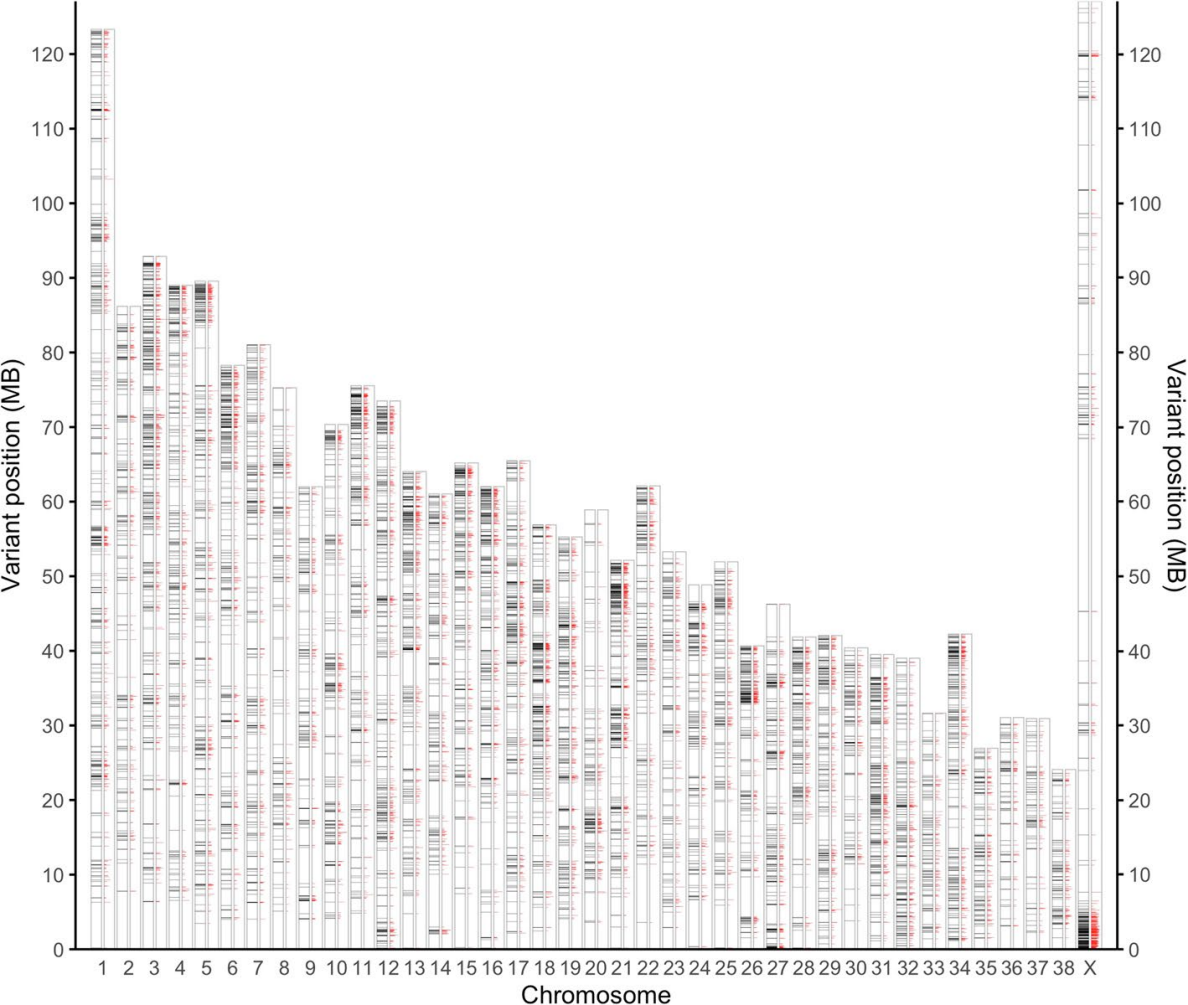
Chromosomal distributions of genetic signatures

We investigated the chromosomal distribution of genome-wide genetic signatures identified from the 76-breed collection. We partitioned the entire genome into contiguous non-overlapping 10 kb-long blocks and analyzed distribution of these blocks by genetic signature density (Fig. 2, Data S9). Overall, 92.91% of genome-wide genetic signatures were enriched in blocks with at least 10 genetic signatures, spanning 69.92% of total genomic regions. The genetic signatures were particularly frequent within the pseudo autosomal region (PAR) of the canine X chromosome (ChrX: 0-6,600,000), where 58.21% (12,530 out of all 21,524) genetic signatures within this region were contained in 238 (36.06%) blocks with more than 40 genetic signatures. On average, each genetic signature in PAR was present in about eight breeds. All 76 breeds were discovered to have genetic signatures in the PAR (Table S9). The enhanced enrichment of genetic signatures within the PAR suggested an excessive selection over genes in this region, which may play an important role in the differentiation of dog breeds.

We partitioned the entire genome into contiguous non-overlapping 10 kb-long blocks and plotted the Whole genome GS distribution across blocks. Y-axis measures the distance to the start of each chromosome (5’ end of the DNA sequence). Each bar was resized proportionally to reflect the relative length of chromosome. The left bar in black of each group indicates the chromosomal distribution of nonoverlapping 10 kb-long genomic blocks with at least 40 breed signatures. Each block was shown as a horizontal black line and mapped to its relative chromosomal position on the bar. The right bar in red of each group indicates the average number of breeds carrying each signature. The length of each red line (scaled by 14.60, which is the maximum average breed number observed) reflects such measurement within corresponding 10 kb-long blocks.

### Genomic regions containing functional breed-specific SNPs, INDELs and STRs indicate genes under extensive selection in specific dog breeds

As it would be valuable to identify the genomic regions enriched for BSGS containing protein-coding genes, we performed analysis of breed-specific long genomic segments, literature surveys and curated public databases for functions and biological pathways (Table S16). We first identified long breed-specific genomic structures comprised of closely located BSGS for the same breed. A total of 696 such structures were identified across 50 different breeds, accounting for 58.99% SNP BSGS, 63.17% INDEL BSGS and 47.68% STR BSGS. We further searched for long stretches of breed-specific genomic segments containing functional BSGS. Fifty-one such breed-specific genomic segments, each containing at least one functional BSGS, were discovered for 19 different breeds (Table 5). The length of these segments varied from 8 kb to 1.3mb, demonstrating high diversity both within each breed and across breeds.

**Table 5:** Breed-specific genomic structures containing functional SNPs

**Breed**	**Chromosomal position**	**Length of breed-specific segments (kb)**	**Predicted effect(s) of functional BSGS**	**Gene(s) harboring functional BSGS**	**BSGS by variant type**				**Average signature density within segments (per kb)**
					**SNP**	**INDEL**	**STR**	**Total**	
Airedale Terrier	chr17:50012842-50334447	321.61	missense variant	C17H2orf78	20	2	1	23	0.07
	chr18:40906402-41128015	221.61	missense variant	LOC541568	14	2	0	16	0.07
	chr19:36987575-37291223	303.65	missense variant	NCKAP5	8	3	0	11	0.04
Akita	chr5:13130523-13821043	690.52	missense variant	ARHGEF12	41	5	2	48	0.07
	chr11:50780958-51865662	1084.70	missense variant	AQP3,NFX1,NOL6	214	47	8	269	0.25
	chr16:46717493-46747972	30.48	missense variant	KLKB1	14	3	1	18	0.59
	chr17:1900616-2086392	185.78	missense variant	EIPR1	180	23	1	204	1.10
	chr20:32827427-33003228	175.80	missense variant	SLMAP	36	13	2	51	0.29
	chr20:45760355-45826036	65.68	missense variant	NIBAN3	20	2	0	22	0.33
	chr20:57465169-57523512	58.34	missense variant	JSRP1	15	8	0	23	0.39
	chr39:2844637-2852948	8.31	missense variant	LOC119863881	34	0	0	34	4.09
Alaskan Malamute	chr3:69753539-70407326	653.79	missense variant	SLC2A9	18	2	0	20	0.03
	chr6:56960224-57238550	278.33	missense variant	BTBD8	15	2	0	17	0.06
	chr13:29471613-29610932	139.32	missense variant	TMEM71	36	8	1	45	0.32
	chr28:10027450-10507275	479.83	missense variant	PIK3AP1	60	10	0	70	0.15
	chr33:25013992-25443707	429.72	missense variant	FBXO40,GOLGB1	17	4	1	22	0.05
Basset Hound	chr6:11567280-12032579	465.30	missense variant	KDELR2	15	4	0	19	0.04
Border Terrier	chr7:44609315-44717971	108.66	missense variant	LOXHD1	37	4	0	41	0.38
Boxer	chr9:5285601-5673185	387.58	missense variant	MYO15B,SAP30BP	63	13	0	76	0.20
	chr9:5976051-6889524	913.47	missense variant	CD300A,OTOP2	81	5	0	86	0.09
Bull Terrier	chr12:42661670-43044269	382.60	conservative inframe deletion	TENT5A	74	19	2	95	0.25
	chr25:27475233-28024366	549.13	missense variant	RP1L1	11	6	0	17	0.03
Bullmastiff	chr3:81428987-81653622	224.64	missense variant	LOC100855743	17	0	1	18	0.08
Chow Chow	chr2:66263373-66419275	155.90	missense variant	C2H16orf78	30	1	0	31	0.20
	chr6:10001047-10033168	32.12	missense variant	ZKSCAN5	14	1	0	15	0.47
	chr6:35660731-36998304	1337.57	missense variant	UBN1	94	14	1	109	0.08
	chr6:37415262-37953252	537.99	missense variant	SLX4	50	13	2	65	0.12
	chr6:39076486-39991490	915.00	missense variant	IFT140	40	4	0	44	0.05
	chr6:40096729-40357537	260.81	missense variant	CAPN15	8	2	0	10	0.04
	chr8:45284725-45472976	188.25	disruptive inframe deletion	SIPA1L1	32	5	0	37	0.20
	chr8:47891946-48095655	203.71	missense variant	LTBP2	20	4	1	25	0.12
	chr12:45073213-45281875	208.66	missense variant	CEP162	34	16	0	50	0.24
	chr21:29910017-30083728	173.71	missense variant	LOC119864893,OR56A9	37	9	1	47	0.27
Collie	chr8:16704547-16837067	132.52	missense variant	CLEC14A	30	2	0	32	0.24
Dalmatian	chr10:70276981-70321406	44.43	missense variant	ANKRD53	9	1	0	10	0.23
Doberman Pinscher	chr16:28937962-29623842	685.88	missense variant	STAR	67	11	2	80	0.12
Keeshond	chr18:15139406-15507417	368.01	missense variant	PUS7	110	22	2	134	0.36
Manchester Terrier	chr12:6091394-6413779	322.39	missense variant	RAB44	23	2	0	25	0.08
Miniature Schnauzer	chr7:31127300-31246535	119.24	missense variant	MAEL	11	3	0	14	0.12
	chr12:56024371-56111012	86.64	missense variant	GPR63	25	2	0	27	0.31
	chr35:25561153-25688479	127.33	missense variant, nonsense variant	LOC488316	13	2	1	16	0.13
Norwich Terrier	chr5:81757408-82203908	446.50	missense variant	CTRL,SLC12A4	56	14	1	71	0.16
	chr5:82436555-82644745	208.19	frameshift variant, missense variant	ZDHHC1,PLEKHG4	50	6	1	57	0.27
	chr18:52446869-52865246	418.38	missense variant	PCNX3,SNX32	17	3	0	20	0.05
	chr31:35605994-35898910	292.92	missense variant	ZBTB21	29	2	0	31	0.11
Rhodesian Ridgeback	chr17:23337069-23518139	181.07	missense variant	TOGARAM2	44	7	0	51	0.28
Samoyed	chr38:22312813-22437410	124.60	nonsense variant	SLAMF8	21	2	0	23	0.18
Sussex Spaniel	chr11:51803380-51998833	195.45	nonsense variant	LOC119873934	23	6	0	29	0.15
	chr15:22956579-23128659	172.08	missense variant	OTOGL	59	14	2	75	0.44
	chr27:5355786-6049814	694.03	missense variant	LMBR1L	34	4	1	39	0.06
	chr30:14802522-15274095	471.57	missense variant	CEP152	104	24	0	128	0.27

Table 5 showed the results of long breed-specific genomic segments that contained functional BSGS. For example, in the Sussex Spaniel, one long genomic segment containing 23 SNP BSGS and 6 INDEL BSGS was found to harbor a nonsense variant, causing premature stop codon in LOC119873934 (40S ribosomal protein S15a-like). In the Samoyed, the nonsense BSGS that severely truncated SLAMF8 was located in a long segment with another 20 SNP BSGS and 2 INDEL BSGS. In the Chow Chow, a 188 kb-long breed-specific segment with 32 SNP BSGS and 5 INDEL BSGS contained three-nucleotide deletions in the SIPA1L1 gene. The deletion would lead to a loss of two consecutive amino acids in SIPA1L1 protein. The functional STR contraction BSGS found in the TENT5A gene of the Bull Terrier within a long Bull-Terrier-specific segment. Such 382-kb-long segment harbored 74 SNP BSGS, 19 INDEL BSGS and 2 STR BSGS, remarking a comprehensive and distinctive genomic structure exclusively owned by the Bull Terrier. A two functional BSGS duo was found to each form a linked BSGS duo with another missense BSGS in the corresponding breed. The first functional duo was found specific to the Miniature Schnauzer, containing one nonsense SNP BSGS and one missense SNP BSGS both within LOC488316 (zinc finger protein 501-like). Other Miniature-Schnauzer-specific BSGS found tagged to this functional duo include 11 SNP, two INDEL and one STR located within the 127 kb-long genomic structure. The other influential functional BSGS duo was observed exclusively by the Norwich Terrier, comprised of one INDEL BSGS causing frame-shift in ZDHHC1 and one SNP BSGS causing amino acid substitution in PLEKHG4 gene. This duo was also found attached to a 208 kb-long Norwich-Terrier-specific genomic structure. Such structure contained 49 SNP BSGS, 5 INDEL BSGS and 1 STR BSGS besides the functional duo. Besides, another two functional duos each comprised of two missense BSGS were also observed in Norwich Terrier. Two missense signatures in CTRL and SLC12A4, respectively, were bound to a 446 kb-long segment located 200 kb upstream the influential the Norwich-Terrier-specific functional duo mentioned before. This segment harbored a total of 71 Norwich-Terrier-specific signatures covering all three types of variants we investigated. The second duo featured one missense BSGS in PCNX3 and one missense BSGS in SNX32, spanning a 418 kb-long-region on chromosome 18. This segment covered another 17 SNP BSGS and 3 INDEL BSGS exclusively present in the Norwich Terrier.

The Akita, Alaskan Malamute and Boxer were also found to have breed-specific long genomic structure containing multiple functional BSGS. One Akita-specific genomic structure harbored a total of three missense BSGS located in the coding regions of AQP3, NFX1 and NOL6, respectively. The entire segment spanned 1.08mb in length, containing 269 BSGS of all three types including 214 SNPs, 47 INDELs and 8 STRs. This is also one of the most significant breed-specific genomic structures we found in terms of length, BSGS number per variant category and functional BSGS count. Notably, the signature density of this segment is 0.25 per kb, which is also on the upper side compared to the median value of 0.16 per kb. The Alaskan-Malamute-specific structure featured one missense variant in FBXO40 and one in GOLGB1. Although signatures in this BSGS stretch were relatively distantly spaced (average signature density 0.05 per kb), they still covered all three types of BSGS which indicated the signature diversity of this region. Lastly, two relatively adjacent genomic segments (chr9:5285601-5673185 and chr9:5976051-6889524) each with a missense BSGS duo were found in the Boxer. The first segment featured two missense BSGS in MYO15B and SAP30BP while the second featured two in CD300A and OTOP2. Both segments contained a relatively high amount of BSGS (76 and 86 in total), providing extensive evidence on selective marks of Boxer over these two long genomic regions.

### Selected validation of nonsense variants by Sanger sequencing in additional dogs

We conducted Sanger sequencing over four nonsense BSGS loci in SLC28A1, MYH16, SLAMF8 and PIBF11 for five additional dogs (Table S12). The results validated, albeit partially, the WGS analyses. The five newly collected dogs included one German Shepherd Dog, one Labrador Retriever, one English Bulldog, one Samoyed and one Golden Retriever. The Sanger sequencing confirmed that A) the Samoyed-specific nonsense locus in SLAMF8, was present in the Samoyed dog and absent in other four dogs, and B) the other three nonsense loci were absent in all five dogs (Table S13). This coincided our findings in the WGS analysis.

## Discussion

Dog breeds have long been a fascinating object for studying population differentiation as the phenotypes are highly homogeneous within each breed while drastically vary across breeds. There is a strong interest in localizing the genetic elements that differentiate dog breeds and contribute to their breed-defining traits. In this study, we assembled a WGS dataset featuring 76 different dog breeds to discover the core genetic signatures that can be stably inherited in each dog breed and comparatively investigated the common and differential genetic signatures across breeds. On the basis of genetic signatures, we constructed a comprehensive genetic variant catalog that captures significant breed-differentiation signatures at the whole genome scale. We exhaustively analyzed all common types of short genetic variants including SNPs, INDELs and STRs, which provided evidence on how dog breeds differentiated from multiple perspectives. Overall, all these signatures are significant by nature as each of them is homogeneously presented in all dogs from the target breeds but are absent in all other dog breeds. Intrinsically the breed-specific signatures of each dog breed represent the unique set of genetic variants that sets each breed apart from all others. This catalog generates abundant information on candidate gene targets behind breed-defining traits as well as uncovered complicated and intertwined evolutionary history of different dog breeds.

Overall, in this study, we searched the whole genome of 412 dogs covering 76 breeds and identified a large variety of breed-specific signatures that were exclusively present in a single breed. In summary, we identified 27,845 SNP signatures in 68 breeds, 4,341 short INDEL signatures in 58 breeds and 604 STRs signatures in 54 breeds. Among them, 143 functional signatures were identified, spanning a total of 30 breeds. Long segments of breed-specific signatures as well as large breed-specific STR expansions were also found within certain genomic regions, revealing genes underwent excessive selection in certain breeds.

The genetic signatures contain rich information about the breeding history of each breed. As the homozygous genetic variants within different breeds can be considered as the results of selection (both natural and artificial), the number of signatures across breeds reflect the relative standing of a breed in its formation process. We propose that, a larger number of breed signatures across the genome, which corresponds to lower within-breed genetic heterogeneity, indicates the relative maturity of a breed from the evolution perspective. On the contrary, a lower number of genetic signatures in turn indicates an early stage that a breed is currently at, showing its active evolving status. Some well-acknowledged ancient breeds with thousand-year breed histories (Table S10) such as the Alaskan Malamute (N_GS_ = 690,487), Chow Chow (N_GS_ = 849,612), Bernese Mountain Dog (N_GS_ = 615,709) and Shin Tzu (N_GS_ = 507,765), were discovered to have a large number of breed signatures.

Eight breeds did not have any breed-specific signatures (Australian Shepherd, Beagle, Bichon Frise, Dachshund, English Springer Spaniel, Golden Retriever, Labrador Retriever and Pointer). Lacking a breed-specific signature might be due to high similarity to a progenitor breed that was included in the analyses. It has been documented that dog breeds were typically descended from a small number of founders and created by crossing closely related individuals [5, 32]. Breeds with short breeding history appeared to have fewer signatures, as can be seen in the Golden Retriever (N_GS_ = 70,907) and German Shepherd (N_GS_ = 105,095) that were both introduced in late nineteenth century. This might reflect modern breeding strategies to produce favorable traits observed by humans during interactions with dogs. Thus, their genomes are still actively evolving in the ongoing processes of selective breeding. However, the year of traceable breed history does not have any overall correlation with the number of breed signatures as many other factors could also influence the genetic background of modern dog breeds. For instance, some ancient breeds that were rebuilt in the post-world-war era such as the Bichon Frise (N_GS_ = 94,122), Maltese (N_GS_ = 205,097) and Vizsla (N_GS_ = 197,891) resemble much younger breeds genetically in respect to the number of breed signatures. Moreover, breeds with high within-breed heterogeneity such as the Dachshund (N_GS_ = 38,490) and breeds with recent admixture history such as the Beagle (N_GS_ = 53,331) also tend to have fewer genetic signatures when compared to breeds emerged during the same era. Similarly, breeds such as the Sussex Spaniel (N_GS_ = 1,396,307), Boxer (N_GS_ = 940,572) and Bull Terrier (N_GS_ = 1,112,485) were found to have considerably higher amount of breed signatures than breeds of similar age, indicating either the underestimated breed history of them or selective pressure imposed on them during the breed formation process. In this sense, the magnitude of breed signatures can both help us recover a less biased breed history and reveal certain event that impacted the formation of modern dog breeds.

The genetic signatures shared by different breeds provided insights on the genetic relatedness between breeds. The fraction of total shared genetic signatures between two breeds among the total genetic signatures of each of the single breeds provided a sensible metric to quantify breed similarities. High genetic-signature-sharing based similarity scores were mostly observed between breeds with high morphological similarities, indicating the unique common evolution history between them. These results were further supported by the identified stretches of breed-pair-specific signatures as showing in Table S3. Furthermore, the genetic signature map also provided information about genetic relatedness for individual dogs not involved in the construction of this signature map. Here we showed a few examples from our applications of this signature map to additional dogs. One Australian Cattle dog (not among the 76 breeds) was shown carrying the highest number of genetic signatures from Border Collie and Australian Shepherd (> 50%), while sharing the lowest number of genetic signatures with the Akita, Bull Terrier and Alaskan Malamute. One Pekingese (not among the 76 breeds) was shown most related to the Lhasa Apso, Shih Tzu, and Tibetan Spaniel, while least related to the Collie, English Bulldog and Boxer in the sense of genetic signatures sharing. Similarly, a French Mastiff (not among the 76 breeds) was shown most related to the Boston Terrier and American Staffordshire Terrier and French Bulldog while least related to the Chinese Shar-Pei and Alaskan Malamute. One rescued dog whose exact breed was unknown appeared to share most genetic signatures with the Labrador Retriever, Beagle, and Dachshund (Table S11).

For BSGS, we showed that the breed distribution appeared to be uneven across breeds, regardless of variant types. Ancient breeds such as the Chow Chow, Alaskan Malamute, Akita, and Sussex Spaniel were found to have a large number of unique signatures in contrast to recently diverged breeds, such as the Golden Retriever and German Shepherd which had no specific signatures at all. Notably, these four ancient breeds ranked as the top four breeds with the highest BSGS discovered in all variant categories. This suggests that these breeds have been genetically isolated from all other breeds for more generations compared to breeds with significantly less BSGS, which reflect the genetic uniqueness of the breed.

Considering the exclusivity of BSGS, they are reasonably linked to phenotypes that are in favor of either artificial or natural selection drives of corresponding breeds. With this purpose, we identified a set of functional signatures with significant biological impacts. We found that gene SLC28A1 was heavily truncated within the Bernese Mountain Dog, which were originally bred in the cold mountain region of Switzerland. Interestingly, this gene has previously been shown to be differentially expressed in Min pigs after cold treatment at the transcript level [33]. This indicates that the SLC28A1 signature might mark the functional adaptation of the Bernese Mountain Dog to the cold climate. We also identified a nonsense BSGS that could severely truncate the SLAMF8 gene in the Samoyed. The SLAMF8 gene is well known for its association with inflammatory bowel disease as indicated by human GWAS [34]. Researchers have also found the knockout of SLAMF8 gene can alleviate arthritis in mice [35]. The Samoyed was originally bred as a sled dog to pull heavy loads for humans, whose utility can be severely hindered by arthritis. In aggregate, this unique signature of the Samoyed seems to be artificially selected to have long-term working durability. In addition, one disruptive inframe deletion in SIPA1L1 gene was found uniquely fixed within the Chow Chow. SIPA1L1 gene functions in regulating synaptic function and maintaining neuronal activities. Functional study has revealed that SIPA1L1 knockout can lead to hyperactivity and enhanced anxiety level in mice [36]. Meanwhile, the Chow Chow have long been used as guarding dogs since ancient China and are well known for their extreme guarding tendencies. These suggest that such signature is a likely breeding result of their utility to human.

## Conclusion

We constructed a high-resolution sequence map for 412 dogs and analyzed the breed-specific genetic signatures for 76 breeds. We identified novel functional BSGS presumably with phenotypic impacts. Four nonsense BSGS were found. SLC28A1 (Solute Carrier Family 28 Member 1) and SLAMF8 (SLAM Family Member 8) were severely truncated in the Bernese Mountain Dog and Samoyed, respectively. PIBF1 (Progesterone Immunomodulatory Binding Factor 1) and MYH16 (Myosin Heavy Chain 16) were partially truncated in the Bull Terrier and Basset Hound, respectively.

Four breed-specific INDEL were found to cause either frameshift or disruptions of codons in four different breeds. The Norwich Terrier and Airedale Terrier carried a frame-shift variant in ZDHHC1 (Zinc Finger DHHC-Type Containing 1) and OR5J2 (Olfactory Receptor Family 5 Subfamily J Member 2) gene, respectively. The Chow Chow carried an INDEL that can cause disruptive in-frame deletion in SIPA1L1 (Signal Induced Proliferation Associated 1 Like 1). The Bernese Mountain Dog carried an INDEL leading to the loss of one codon in CENPU (Centromere Protein U).

Eighteen breeds were found to carry novel breed-specific SNP-clusters in at least 10 contiguous breed-specific SNPs in the short genomic regions of about 1 kb in length. These breeds included the Airedale Terrier, Akita, Alaskan Malamute, Border Terrier, Cairn Terrier, Chow Chow, Collie, Doberman Pinscher, Field Spaniel, Irish Wolfhound, Keeshond, Manchester Terrier, Miniature Schnauzer, Norwich Terrier, Rhodesian Ridgeback, Rottweiler, Sussex Spaniel, and Tibetan Terrier.

Breed-specific STR expansions were found, in which the Akita, Alaskan Malamute, Chow Chow and Sussex Spaniel carried the highest numbers of such expansions. When compared to other dog breeds, the Alaskan Malamute was found to carry significantly long STR expansions around three gene regions, SGIP (SH3 Domain GRB2 Like Endophilin Interacting Protein 1), DPP6 (Dipeptidyl Peptidase Like 6) and EAF1 (ELL Associated Factor 1). The Akita carried a long STRs expansion upstream the ABRA (Actin Binding Rho Activating Protein) gene. Besides, the Akita were also found to have three different breed-specific STRs expansions in the gene region of ZCCHC10 (Zinc Finger CCHC-Type Containing 10). Interestingly, ZCCHC10 also contained breed-specific SNP-clusters in this breed.

Together, we found 15 signature genomic regions with all three types of BSGS (i.e., SNP-clusters, INDELs and STRs) in seven breeds (Akita, Alaskan Malamute, Chow Chow, Field Spaniel, Keeshond, Shetland Sheepdog, Sussex Spaniel). Notably, the Keeshond and Sussex Spaniel each had a signature set covering the genes resulting in an amino acid change in PUS7 (Pseudouridine Synthase 7) and OTOGL (Otogelin-Like Protein) proteins, respectively.

According to the similarity scores based on the SNP data, the most genetically similar pairs were the Collie vs. Shetland Sheepdog and Akita vs. Chow Chow. The least genetically similar breed-pairs were the Dachshund vs. Labrador Retriever and Dachshund vs. Golden Retriever. Surprisingly, the English Bulldog were genetically distant to Chinese Shar-Pei (a low similarity score between the two) despite their similarities in observable phenotypes, which, in turn, coincides our hypothesis that there might be unknown phenotypes yet to be uncovered that distinguished the two breeds.

In conclusion, every dog breed is genetically related to at least one other breed at various degrees. The BSGS map is a high-resolution genetic atlas that quantitatively distinguishes the breeds of dogs and pinpoints previously unknown genetic markers that are specific to a single breed. The exclusivity of BSGS further provided valuable information on linking certain breed-defining traits to breed-specific genetic variants. Importantly, the approach we employed can be easily generalized to other species besides dogs, as selecting the right genetic backgrounds of the breeds or strains of the animal models has always been one of the most crucial yet unsolved puzzles in the research field of medical science.

## Methods

### Dog sample collection and genomic DNA extraction

We collected the leftover blood samples of 28 dogs through our collaboration with local veterinary clinics. Experienced veterinarians drew blood from the front arm of participating dogs with dog owners’ consent during the medical care of dogs. Sample and breed information was collected from the dog owners during their visits (S4 Data). All the blood samples were treated with anticoagulant to prevent clotting during the transportation and storage process. We extracted genomic DNA from each sample using QIAGEN DNA Blood Mini kit following the standard protocol. Sample quality control was carried out on Fisher NanoDrop as well as PicoDrop to make sure the DNA concentration and purity level fulfill the requirement of WGS library preparation (total gDNA > 0.5ug, 1.8 ≤ A260/A280 ≤ 2.0). Otherwise, we repeated the extraction on additional blood samples until the quality standards were met.

### Next generation whole genome sequencing

Sample quality controls (QC): Yale Center of Genome Analysis (YCGA) whole genome sequencing (WGS) pipeline starts with stringent quantification and quality control of the received samples. Samples delivered to YCGA are immediately entered into WikiLIMS with the provided sample identifiers. Entrance of the samples to the database generate and assign a second database identifier unique to each submission and sample (sample tracking number). This is followed by standard quantity, quality and purity assessments via determination of the 260/280 nm for values of 1.7–2.0, and 260/230 absorbance ratios for values ≥ and 1% agarose gel electrophoresis to ensure that the gDNA is neither degraded nor displays RNA contamination. Combination of PCR-free library preparation and sequencing on patterned flow-cells of Novaseqs, makes quantification of the starting gDNA of paramount importance. To that end, all samples undergoing PCR-free library preparation will also be quantified using a fluorometric method by Qubit (ThermoFisher Scientific Part#Q33226) for proper assessment of double stranded DNA concentration.

Library Preparation: 0.5ug of well quantified gDNA is undergoing enzymatic fragmentation, end-repair and “A” base in a single reaction using Lotus DNA Library Prep kit(IDT, Part#10001074. The adapters with appropriate dual multiplexing indices, xGen UDI-UMI Adapters (IDT, Part #10005903), are then ligated to the ends of the DNA fragments for hybridization to the flow-cell for cluster generation. Size of the final library construct is determined on Caliper LabChip GXsystem and quantification is performed by qPCR SYBR Green reactions with a set of DNA standards using the Kapa Library Quantification Kit (KAPA Biosystems, Part#KK4854). Size and concentration values will be entered into the WikiLIMS database for the sequencing team’s use for appropriate flow-cell loading.

Flow Cell Preparation and Sequencing: Sample concentrations are normalized to 2 nM and loaded onto Illumina NovaSeq 6000 flow cells at a concentration that yields at least 700Gbp of passing filter data per lane. Loading concentration for WGS libraries has been optimized to maximize both well occupancy and unique read output while limiting duplicates associated with patterned flow cell technology. Samples are sequenced using 151 bp paired-end sequencing reads according to Illumina protocols. The 10 bp indexes are read during additional sequencing reads that automatically follow the completion of read 1. Data generated during sequencing runs are simultaneously transferred to the YCGA high-performance computing cluster. A positive control (prepared bacteriophage Phi X library) provided by Illumina is spiked into every lane at a concentration of 1% to monitor sequencing quality in real time.

Signal intensities are converted to individual base calls during a run using the system’s Real Time Analysis (RTA) software. Base calls are transferred from the machine’s dedicated personal computer to the Yale High Performance Computing cluster via a 1 Gigabit network mount for downstream analysis. Primary analysis—sample de-multiplexing and alignment to the human genome—is performed using Illumina’s CASAVA 1.8.2 software suite. The data is returned returned to the user if the sample error rate is less than 2%. Data is retained on the cluster for at least 6 months, after which it is transferred to a tape backup system.

### WGS raw data quality control and assembly

Whole genome sequencing of 23 lab-collected gDNA samples was performed on Illumina Novaseq 6000 platform in pair-ended mode detailed as described above. Each sample was sequenced using 151 bp reads with 30 × average genome coverage. We additionally downloaded the whole genome sequencing data of 429 dogs from 95 breeds (S5 Data) from sequencing read archive (SRA). We carried out initial quality control of raw sequencing data using FastQC v0.11.9 [37]. We removed all detected adapter sequences and low-quality sequences using Trimmomatic v0.39 [38] while keeping reads with at least 50 bp after the trimming. The quality of trimmed samples was checked again to ensure all low-quality parts had been successfully removed. We aligned all the QC-passed reads to the current representative dog reference genome *ROS_Cfam1.0* (https://www.ncbi.nlm.nih.gov/assembly/GCF_014441545.1/) for each sample using BWA v0.7.17 [39] and removed duplicated reads using Samtools v1.12 [40]. Alignment results for each sample were sorted by chromosomal coordinate and stored in BAM format to save storage space. We used Samtools to count the per-site sequencing depth across the genome. We then calculated and reported the average sequencing depth of 38 autosomes and the X chromosome for all samples collected.

### Variant discovery for each dog WGS data

We then followed the GATK v4.2.0.0 [41] germline short variant discovery best practice pipeline to generate variant dataset by jointly calling the genomic variants of all 452 dogs. We applied a recommended hard filter to keep biallelic SNPs with high credibility using GATK flags “QD < 2.0”, “QUAL < 30.0”, “SOR > 3.0”, “FS > 60.0”, “MQ < 40.0”, “MQRankSum < -12.5” and “ReadPosRankSum < -8.0”. SnpEff v5.0e [42] was used to annotate the discovered variants and predict the potential variant impact using transcript data on *ROS_Cfam1.0* in NCBI release 106.

### Initial assignments of dog breeds

Since the correct breed label is crucial to our main analysis, we conducted a phylogenetic analysis on all 451 dogs initially enrolled in our study. The distance matrix was built based on the pairwise identity-by-state (IBS) value calculated over pruned autosomal variants. We built the phylogenetic tree using R package phytools [43] and removed samples that were assigned to clades of other breeds (Fig S3a). We removed a total of 8 samples with potentially erroneous clade assignment. Samples from breeds whose breed sample sizes were less than three were further removed. Finally, a total of 412 samples from 76 breeds remained qualified for the breed-based analysis (Fig S3b).

### Unsupervised machine learning to discover GS and BSGS

Starting here, we developed a suite of C +  + based computational programs for the purpose of discovery and analyses of genome-wide genetic signatures. To discover differences and similarities of variants between dog breeds, we calculated the breed variant frequency (BVF) using the number of dogs carrying the variant alleles on both chromosomes divided by the total number of dogs with solid allele type calls for each breed (the proportion of dogs with homogeneous variant allele type for each breed). STR were identified from the candidate multi-allelic loci. Only loci with STR of a single type of repetitive units were involved in the discovery process. Repetitive units were identified via exhaustive comparison between all allele types at a given locus. We characterized STR alleles into either high-repeat group and low-repeat group based on the distribution of repeat counts at the locus. A standard K-mean algorithm was employed to automatically find the classification boundary using the lowest repeat count and highest repeat count at each locus as the initial centroids of two groups. We examined the allele depth of each variant call and only included samples whose allele depth was above a certain threshold at the given locus. The minimal depth threshold is 10 for autosome variants and 5 for X chromosome variants. For identified STR loci with multiple alleles, the total effective allele depth was calculated as the sum of depth of final called allele types. Dogs with unsolid allele calls at a given site were neither counted into the nominator nor the denominator of such equation during the frequency analysis at that site. Considering that the reference dog itself might carry some unique variants, we excluded variants that were presented in all the included dogs from the candidate variant pool. We defined a breed as valid breed at a given variant locus if at least three dogs from the breed are with solid allele calls. Based on previous quality metrics, we identified a variant as the breed genetic signature if such variant has a BVF ≥ 0.9 in any valid breed. In the meanwhile, we identified a variant as the breed-specific genetic signature (BSGS) to a certain breed if such variant has a BVF≥0.9 in the target breed and BVF ≤ 0.1 in all other valid breeds. We exhaustively searched for the genetic signatures and BSGS regarding both the variant allele and reference allele in separate runs and combined the scanning results together. Lastly, we examined the total effective sample size during the discovery of each BSGS and genetic signatures and removed those discovered with relatively small number of samples. For both genetic signatures and BSGS, we filtered out those discovered with less than 300 samples from all effective breeds not carrying the genetic signatures or BSGS. The original scanning results for BSGS and genetic signatures were reported in S3 Data and S6 Data, respectively.

### Computational discovery of BSGS segments

All identified BSGS and breed signatures were summarized by breed and by annotation flag. Nonsynonymous signatures were extracted separately and later summarized by breed. The BSGS stretches were detected using two different schemes for different target properties. We counted the number of BSGS by breed and by genomic region (separate by different genes). Genomic regions with at least 5 BSGS from the same breeds were reported as they reflected gene functional regions with a high amount of BSGS (S2 Data). Alternatively, we also designed a sliding window to scan through the genome-wide BSGS for high-density segments. The minimal size of the window was set to 10 BSGS to avoid being trapped into extremely short local BSGS segments (e.g., two or three adjacently positioned BSGS). We tracked the average BSGS density within each sliding window and reported the longest BSGS stretches with a density of at least 10BSGS/kb.

### Analysis of BSGS and genetic signatures shared between breed-pairs

We additionally scanned for genetic signatures (both BSGS and genetic signatures) shared by multiple breeds to investigate the signature similarity between them. We summarized the detailed information of genetic signatures exclusively observed in two breeds in Table S3. For genetic signatures, we counted the number of signatures showing up in each single breed and breed-pair, regardless of the exclusivity of signatures. By this method, a variant that is homogeneous in three breeds will be counted towards all three breeds and three breed-pairs. The results were filled into a 76 by 76 matrix, with the diagonal elements indicating the number of signatures discovered within each breed and the off-diagonal elements indicating the number of common signatures between two breeds (Table S6). We further calculated the signature similarity between two breeds by dividing the number of common breed signatures by the geometric average of the number of signatures discovered in each of the two breeds. The highest and lowest unstandardized signature similarity scores were transformed into 1 and 0 after the standardization. The larger the similarity score between two breeds, the higher proportion of genetic signatures the two breeds shared with each other. We inferred the breed relationship of individual dogs by calculating the allele similarity between each sample and the genetic signatures of each of the 76 breeds across all 3,892,182 genetic signatures loci. For a given genetic signature with a variant (when being compared to the reference genome) allele type, a sample was considered 0%, 50% and 100% carrying the genetic signature of a target breed if it had homogeneous reference, heterozygous and homogeneous variant allele type, respectively. We summed up the weighted genetic signature carrying score for each of the 76 breeds and then divided the sum by the total number of genetic signatures discovered from each corresponding breed. We generated a list of per-breed genetic-signature-sharing percentages between the target sample and each breed within our collection to indicate the individual ancestry information (S7 Data). The higher fraction of genetic signatures a sample shared with a breed, the higher the relatedness it is to the breed. These metrics can be used to infer the breed of a dog, especially for those from the 76 breeds covered by the genetic signature discovery dataset.

### Overlapping SNP BSGS, INDEL BSGS and STR BSGS

Since SNP BSGS, INDEL BSGS and STR BSGS were drawn from different pools of genomic variants which required different QC and categorization procedures (bi-alellic SNPs, bi-allelic INDELs, multi-allelic INDELs), the discovery process was relatively independent to each other overall. To analyze genomic structures potentially comprised of all three types of BSGS, we first pooled them together. To do so, we prepared filtered SNP BSGS, INDEL BSGS and STR BSGS results to be sorted by chromosome coordinates while only keeping positional, annotation and breed information for each BSGS. We later put each BSGS list into an independent queue and kept track of the foremost elements within each queue. At each time, the element with smallest chromosome coordinate was removed from the original queue and put into the merged list. If multiple BSGS with the same chromosome coordinate but a different BSGS type was found at the same time, they were merged into one query. Throughout the merging process, none of such conditions was found.

### Identification of breed-specific genomic structures comprised of functional BSGS

After obtaining the merged BSGS catalog, we first grouped all types of BSGS by breed while maintaining their chromosomal positions in sorted order. We then traversed the BSGS list by breed and chromosome to identify breed-specific genomic structures. Such structures were defined as the longest BSGS stretches that met the following conditions: 1) adjacent BSGS within the same structure located no more than 100 kb away from each other and 2) each stretch contained at least 10 BSGS. We set the maximum distance between two BSGS as 100 kb for the purpose of maintaining contiguity of each breed-specific structure while taking account of naturally long haplotype of dog breeds. During the scanning process, we kept track of pre-annotated BSGS effect flags and output genomic structures harboring BSGS with protein changing effects. Targeted functional BSGS flags included “missense_variant”, “stop_gained”, “frameshift_variant”, “disruptive_inframe_deletion”, “disruptive_inframe_insertion”, “conservative_inframe_deletion” and “conservative_inframe_insertion”. We also kept track of the composition of BSGS within each qualified breed-specific genomic structure as well as the average variant density within the structure. All these relevant statistics were reported along with each structure identified.

### Spatial distribution analysis of genetic signatures

We partitioned the reference genome into contiguous 10 kb-long blocks and investigated the chromosomal distribution of breed signatures across such block. We counted the number of identified genetic signatures, average genetic signature density and average number of signature-carrying breeds per locus within each block. We stratified the number of such 10 kb-blocks by average genetic signature density (1 genetic signature per KB) for each breed. We sequentially traversed each block to investigate the overall genetic signature distribution across the genome. We calculate the average signature density regardless of the breed for each block and identified the blocks with relatively high density (≥ 40 genetic signatures per KB). Along with it we used the average number of genetic-signature-carrying breeds to reflect the breed genetic signatures diversity of each block. We additionally plotted the high-density genetic signature blocks to highlight genomic regions with large number of genetic signatures as indicated in Fig S1.

### Integration of efficient computational algorithms for genome-wide genetic signature discovery

During the analysis of the dog WGS data, we designed a fast and efficient C +  + based command-line tool to conduct genome-wide screening for variants with certain population frequency constraints. The tool takes a standard Variant Call Format (VCF) variant file and a sample label file as inputs. It then outputs a list of variants that satisfy a series of highly customizable constraints.

Specifically, the tool has three main modules, namely a quality filter, a variant selector, and a population frequency analyzer (Fig S5). The quality filter filters out variant calls with relatively low certainty (e.g., low total allele depth or ambiguous genotype calls) based on the sequencing depth of each individual at a given site. Since the sequencing quality of a given sample could vary from site to site, this filter evaluates the variant quality of each sample at a per-locus basis rather than at the whole-genome scale which maximally maintains samples with high-quality variant calls for the frequency analysis. The variant selector can analyze the positional information and specific annotation information (if applicable) of each variant to allow users to specifically target a group of variants of their interest. For example, the selector can flexibly focus on variants within a certain genomic region or variants of a certain type (e.g., premature stop codon variants and amino-acid changing variants as analyzed in the previous sections) or even variants with more detailed characteristics (e.g., point variants with Glu to Arg transversion or premature stop codon variants with at least 10% of protein sequence lost). With this module users can either do a genome-wide scan for certain types of variants or examine amino-acid changing variants around a previously highlighted locus. The population frequency analyzer, as the kernel module of this tool, can efficiently analyze the population frequency of pre-selected candidate variants within flexibly defined sample groups. A sample group can either contain one or more individually-specified samples or pre-labeled populations. With additionally provided logical expression on frequency threshold for each sample groups, the analyzer can curate target variant sites and output variants that satisfy all the frequency constraints (e.g., frequency ≥ 0.9 in one population and ≤ 0.1 in the other or frequency = 1.0 in one sample and frequency = 0.0 in one population, etc.).

The tool was made to be highly flexible as users can customize all the three important variables in the analysis, including: 1) the target variants of interest; 2) the samples taken into each comparison group; and 3) the frequency constraints used to prioritize variants. Besides, with all the three kernel modules thread up seamlessly, this tool allows users to do quality control, variant selection and population specific variant discovery in one-click starting from the widely accessible VCF files without additional data formatting steps. Although the tool was initially designed to study pedigree dogs as we showcased in previous sections, it can also be effectively applied to other study populations (e.g., humans and viruses) or scenarios (e.g., cases vs. controls) with flexible parameter settings.

### Performance benchmark/evaluation of the integrative GS discovery tool

We additionally implemented multi-threading methods to boost computational performance to handle terabytes of the raw WGS data. We benchmarked three main analytic modules which aimed to discover breed-enriched, breed-unique and breed-pair unique variants, respectively, on the discovery dataset of 412 dogs (Fig S6a). With 10 cores invoked for the variant discovery, the tool scanned through around 29 million variants and finished each of the three analyses in around 10 min. Furthermore, the tool had a very low memory usage throughout the benchmark process (1.52G for breed-enriched variant discovery, 1.37G for breed-unique variant discovery and 1.44G for breed-pair unique variant discovery in average), which means it can operate on personal computers without the high requirement of computing hardware. Moreover, the per-core efficiency was maintained at a relatively high level when the number of cores involved increased (Fig S6b). This allows users of this tool to quickly traverse large WGS datasets and conduct genetic pattern discovery analyses without worrying about sacrificing efficiency.

We benchmarked the tool on a server with Intel Xeon E5-2660 v3 CPUs. The dataset used for the benchmark contains a total of 29,703,668 variants (all bi-allelic). The entire dataset uses 363 GB storage space. We timed each variant discovery analysis using an internal clock implemented in the tool. The average memory usage data was obtained from the Slurm (job scheduler of the server) report. We calculated the per-core efficiency by taking the proportion of average per-core processing speed of each analysis and the highest average per-core processing speed observed during the benchmark.

## Supplementary Information

**Additional file 1: Fig S1.** The number of the nonsense genetic signatures (GS) for each breed. The y-axis represents the number of nonsense variants in GS represented by dots. The x-axis corresponds to each of the 76 breeds in the alphabetical order. **Fig S2.** Histogram showing the breed sample size distribution. The histogram showed the sample size within each valid breed enrolled in the GS discovery process. The minimal breed sample size was capped at three as shown in the histogram. **Fig S3.** Phylogenetic tree showing the breed membership of samples in the collection. Each sample was colored based on its breed label. Samples from the same breed were marked with the same color. a) Tree A includes 451 pure-bred dogs from 97 breeds in the collection. b) Tree B includes 412 pure-bred dogs from 76 valid breeds after removing potential wrongfully labeled samples. **Fig S4.** Pairwise genetic signature sharing between all 76 breeds. The 76 by 76 grid plot indicates the genome-wide GS sharing for all breed-pairs. The upper triangular part shows the standardized GS sharing score (See Methods). All scores were scaled between 0 and 1 with the darkest grid representing the highest GS sharing between Shetland sheepdog and Collie. The lower triangular part shows the absolute number of GS discovered across the genome. Similarly, the darker grid color indicates larger number of GS shared between two breeds. **Fig S5.** General pipeline of the population-frequency based variant analyzing tool. The flowchart showing the general workflow of the population frequency. **Fig S6.** Performance benchmark of the variant analyzing tool. a) Line plot showing the real-world run-time of using this tool to discover GS, BSGS and breed-pair unique shared GS in the dataset of 412 samples, when different number of CPUs were provided. b) The relative per-CPU efficiency for each of the three analyses when different number of CPUs were provided.

**Additional file 2: Table S1.** Breed-specific SNP distribution by breed and by variant category. **Table S2.** Genomic regions with at least 40 breed-specific SNP signature from the single breed. **Table S3.** Detailed information of SNPs exclusively shared by breed-pairs. **Table S4.** Breed-specific INDEL distribution by breed and by variant category. **Table S5.** Breeds with breed-specific large STR expansions. **Table S6.** SNP based genetic signatures distribution across 76 breeds. **Table S7.** Breed similarity matrix based on shared SNP signatures. **Table S8.** Breed-pair list sorted by SNP signature similarity score. **Table S9.** Number of SNP signature within X chromosome pseudo autosomal region. **Table S10.** Breed history information of the 76 breeds. **Table S11.** GS-based similarity analysis of the 23 dogs. **Table S12.** Information of four nonsense BSGS loci for validation. **Table S13.** Sanger sequencing results for five additionally collected samples. **Table S14.** Correlation between SNP, INDEL and STR based GS similarity scores. **Table S15.** Top five breeds similar to French Bulldog according to SNP based GS similarity score. **Table S16.** Biological pathway annotations of highlighted breed-specific functional structures.

**Additional file 3: Data S1.** Genomic regions with at least 5 SNP BSGS from a single breed. This file contains detailed information on genomic segments with at least 5 SNP BSGS from a single breed. Segments were grouped and sorted by breed.

**Additional file 4: Data S2.** SNP GS discovered across the genome. This file contains information on all genome-wide SNP GS discovered from 76 valid breeds.

**Additional file 5: Data S3.** Basic sample information of the 23 dogs from the lab collection. This file contains basic sample information of newly sequenced dogs.

**Additional file 6: Data S4.** Basic sample information of 429 dogs from the Sequence Read Archive (SRA). This file contains basic sample information of dogs whose WGS data was downloaded from SRA.

**Additional file 7: Data S5.** All SNP BSGS discovered across the genome. This file contains information on all genome-wide SNP BSGS discovered from 76 valid breeds.

**Additional file 8: Data S6.** GS-based breed composition of 452 dogs. This file contains GS-carrying percentages of five most and least similar breeds for each individual dog involved in this study.

**Additional file 9: Data S7.** All INDEL BSGS discovered across the genome. This file contains information on all genome-wide INDEL BSGS discovered from 76 valid breeds.

**Additional file 10: Data S8.** All STR BSGS discovered across the genome. This file contains information on all genome-wide STR BSGS discovered from 76 valid breeds.

**Additional file 11: Data S9.** Detailed list of 10-kb-long genomic block with enriched SNP GS signals. This is the summarized data used for generating Fig 2. Information on genes overlapped with each highlighted 10kb block was presented in the last column.

**Additional file 12: Data S10.** Detailed list of SNP, INDEL and STR based GS similarity scores for each breed. This zip file contains 76 text files each corresponds to one valid breed involved in this study. Each text file contains detailed pairwise similarity scores calculated using genome-wide SNP, INDEL and STR GS.

## Data Availability

All data are contained in the manuscript, the supplementary file, or submitted to SRA. Accession number of previously generated WGS data can be found in Data S4. The raw whole genome sequencing data generated by the Hoh lab is available in the Sequencing Read Archive repository, Bioproject accession number PRJNA952529.
