# Supplementary material for: Systematically identifying genetic signatures including novel SNP-clusters, nonsense variants, frame-shift INDELs, and long STR expansions that potentially link to unknown phenotypes existing in dog breeds
Source: BMC Genomics. 2023 Jun 5;24:302. doi: 10.1186/s12864-023-09390-6 (PMC10240460; doi:10.1186/s12864-023-09390-6)
Supplement: Supplementary file 1 — Additional file 1: Fig S1. The number of the nonsense genetic signatures (GS) for each breed. The y-axis represents the number of nonsense variants in GS represented by dots. The x-axis corresponds to each of the 76 breeds in the alphabetical order. Fig S2. Histogram showing the breed sample size distribution. The histogram showed the sample size within each valid breed enrolled in the GS discovery process. The minimal breed sample size was capped at three as shown in the histogram. Fig S3. Phylogenetic tree showing the breed membership of samples in the collection. Each sample was colored based on its breed label. Samples from the same breed were marked with the same color. a) Tree A includes 451 pure-bred dogs from 97 breeds in the collection. b) Tree B includes 412 pure-bred dogs from 76 valid breeds after removing potential wrongfully labeled samples. Fig S4. Pairwise genetic signature sharing between all 76 breeds. The 76 by 76 grid plot indicates the genome-wide GS sharing for all breed-pairs. The upper triangular part shows the standardized GS sharing score (See Methods). All scores were scaled between 0 and 1 with the darkest grid representing the highest GS sharing between Shetland sheepdog and Collie. The lower triangular part shows the absolute number of GS discovered across the genome. Similarly, the darker grid color indicates larger number of GS shared between two breeds. Fig S5. General pipeline of the population-frequency based variant analyzing tool. The flowchart showing the general workflow of the population frequency. Fig S6. Performance benchmark of the variant analyzing tool. a) Line plot showing the real-world run-time of using this tool to discover GS, BSGS and breed-pair unique shared GS in the dataset of 412 samples, when different number of CPUs were provided. b) The relative per-CPU efficiency for each of the three analyses when different number of CPUs were provided. [file 12864_2023_9390_MOESM1_ESM.pptx]

## Slide 1
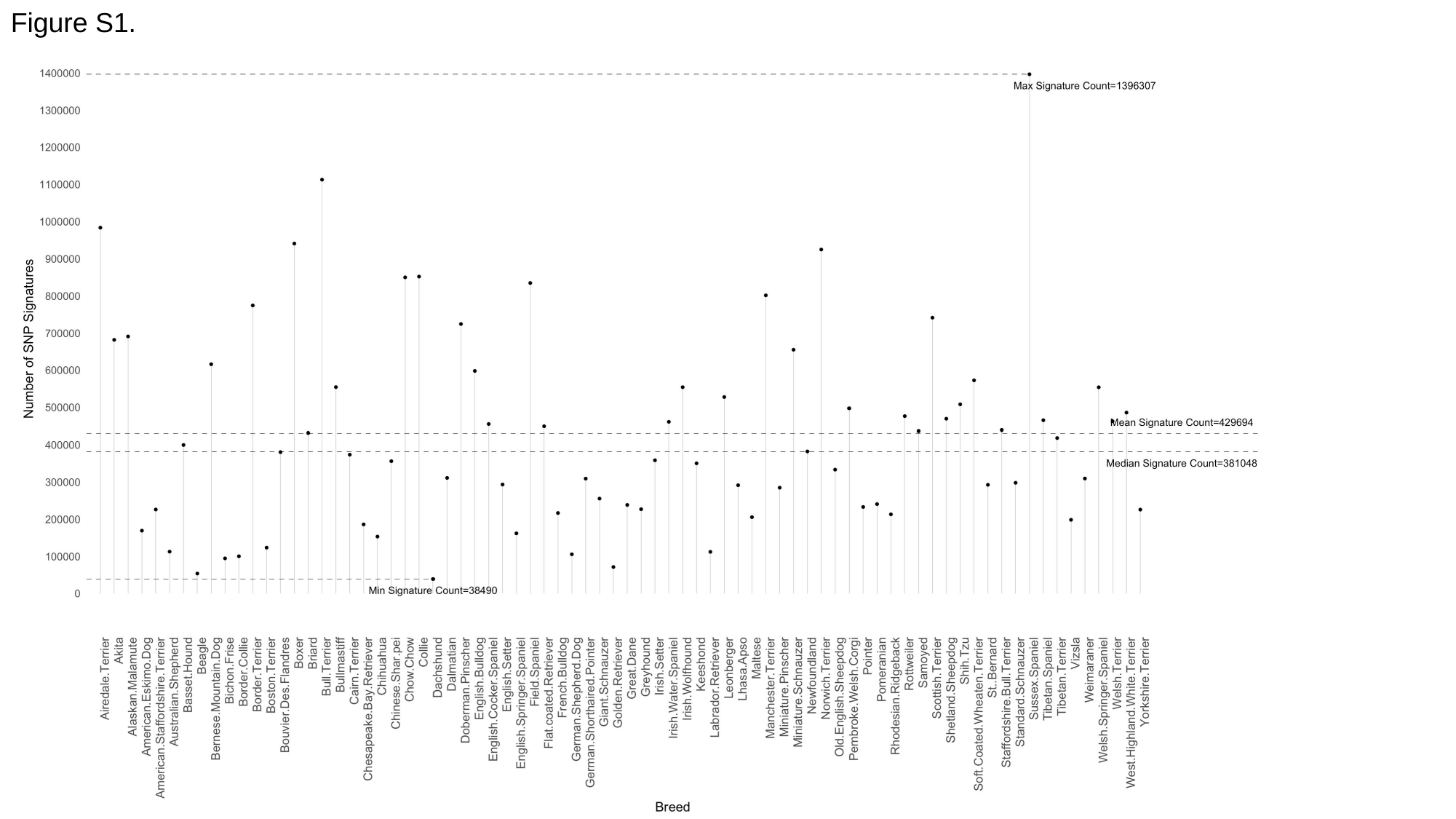

Figure S1.

## Slide 2
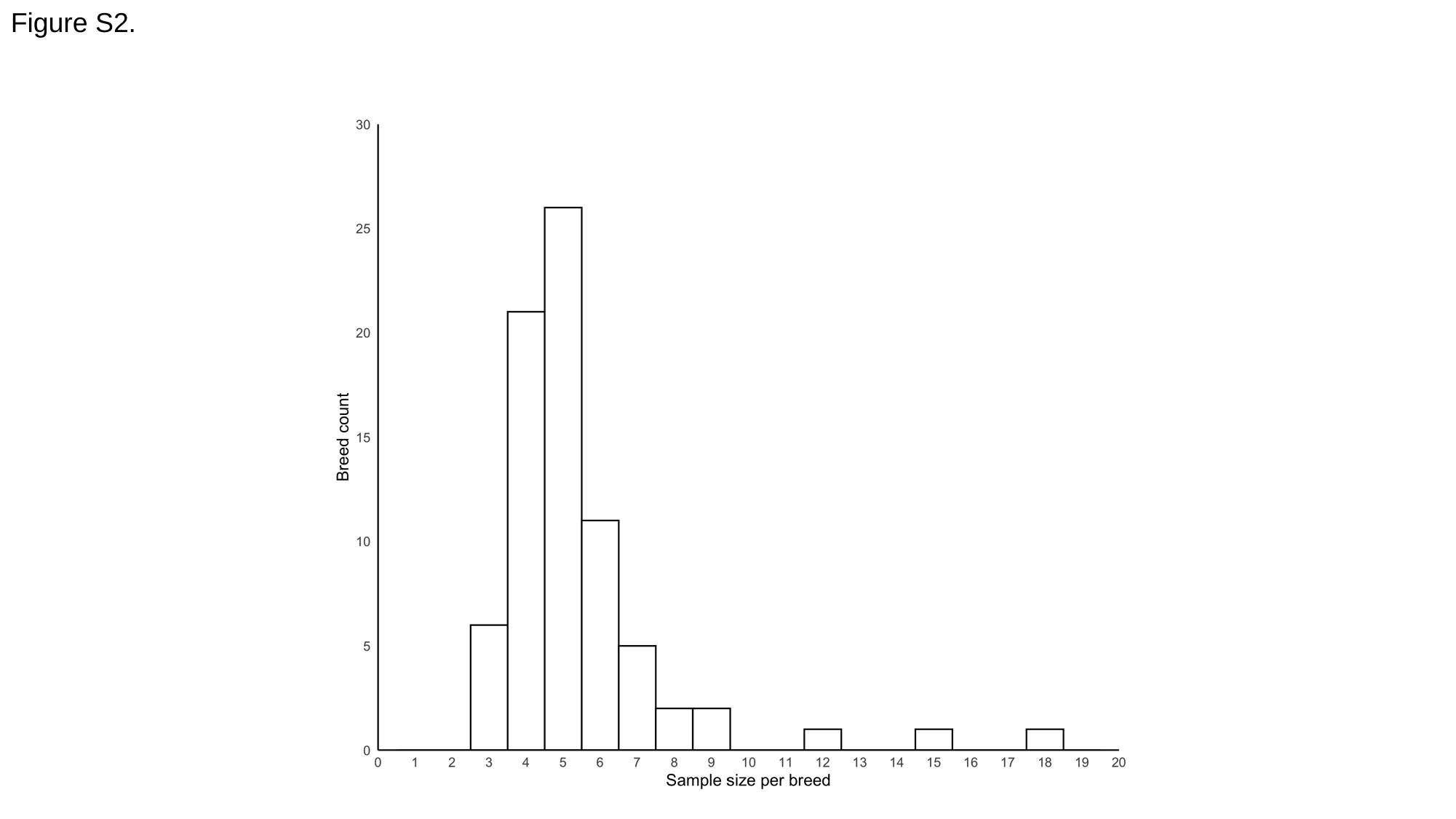

Figure S2.

## Slide 3
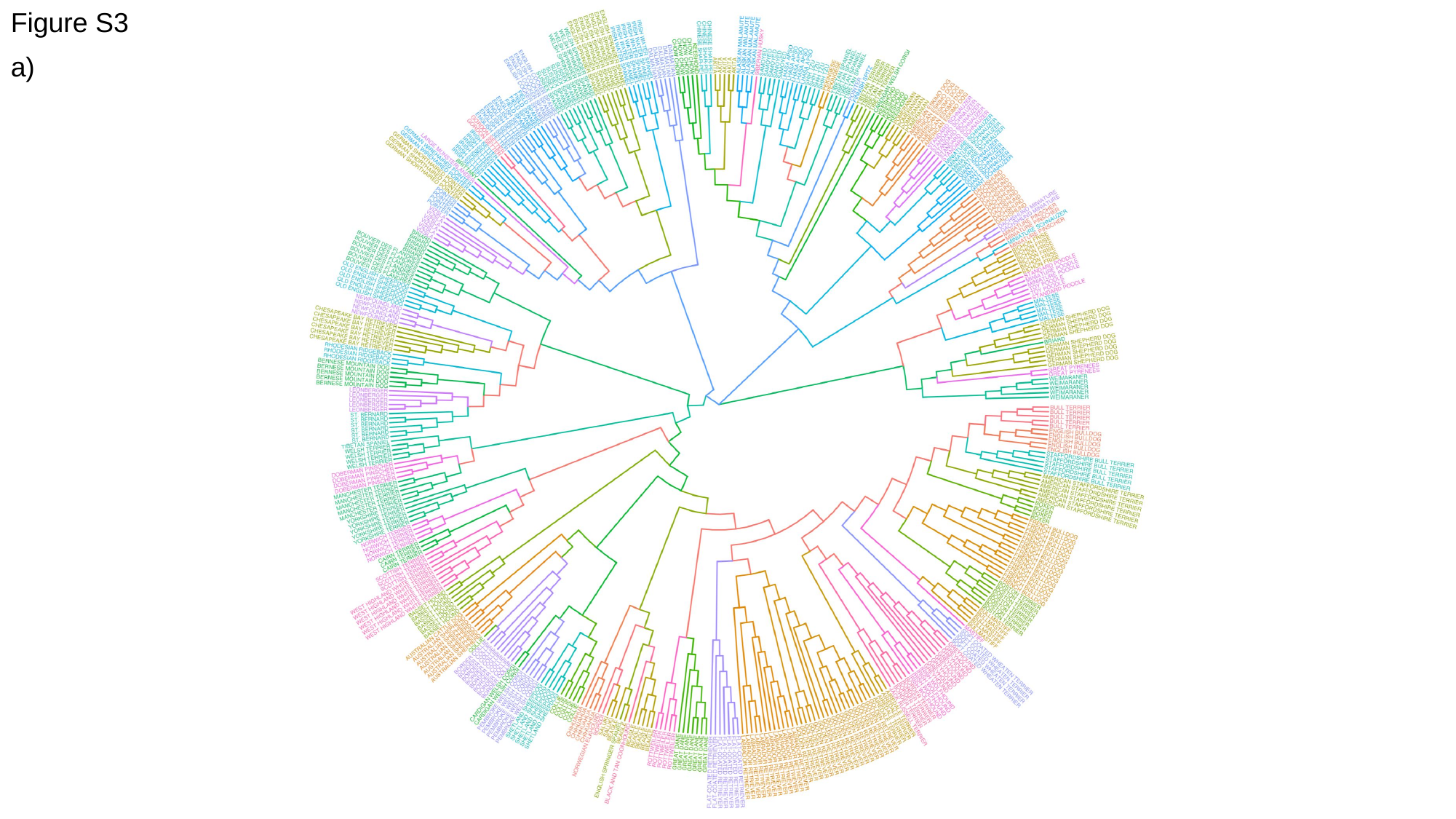

Figure S3
a)

## Slide 4
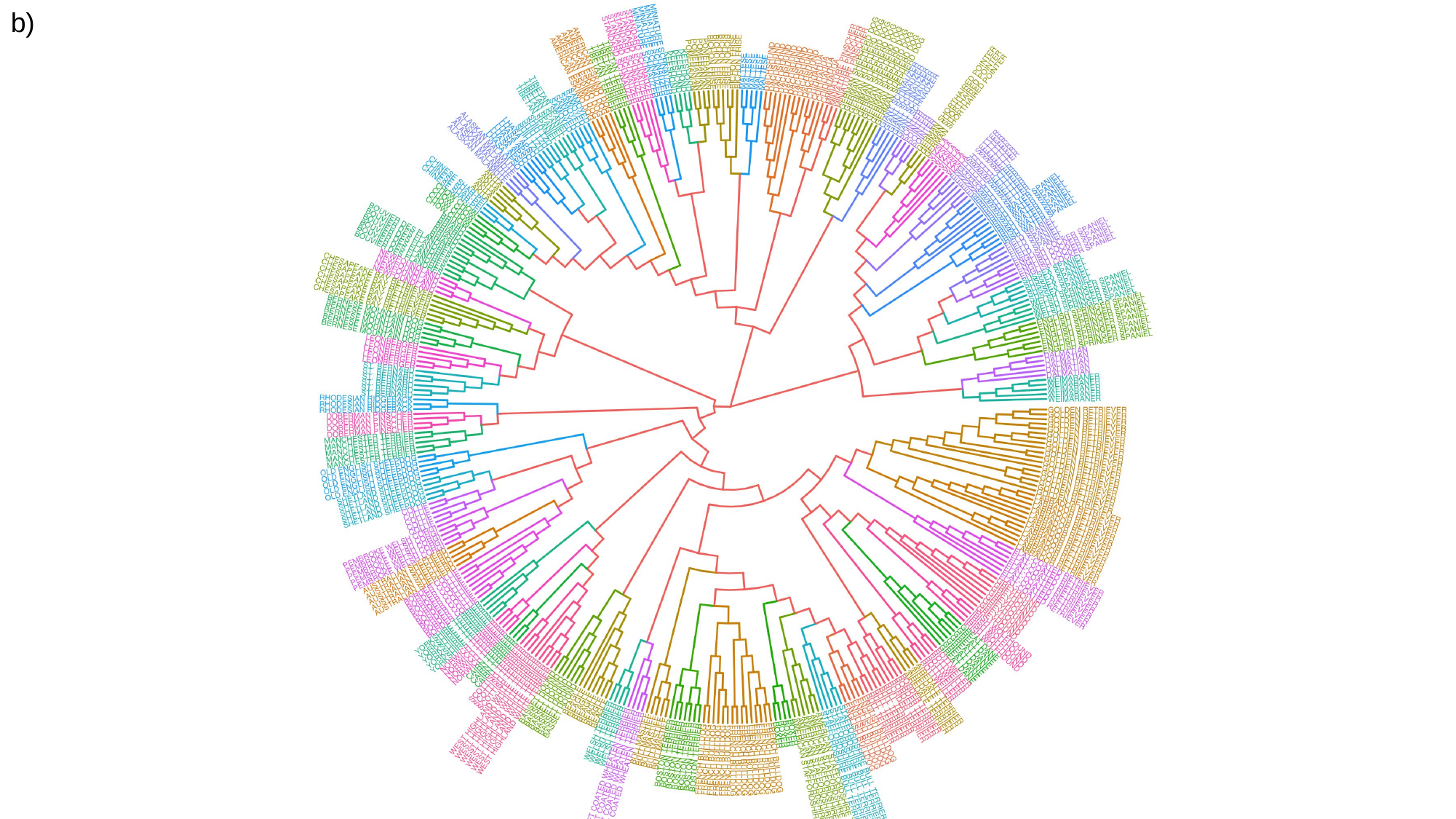

b)

## Slide 5
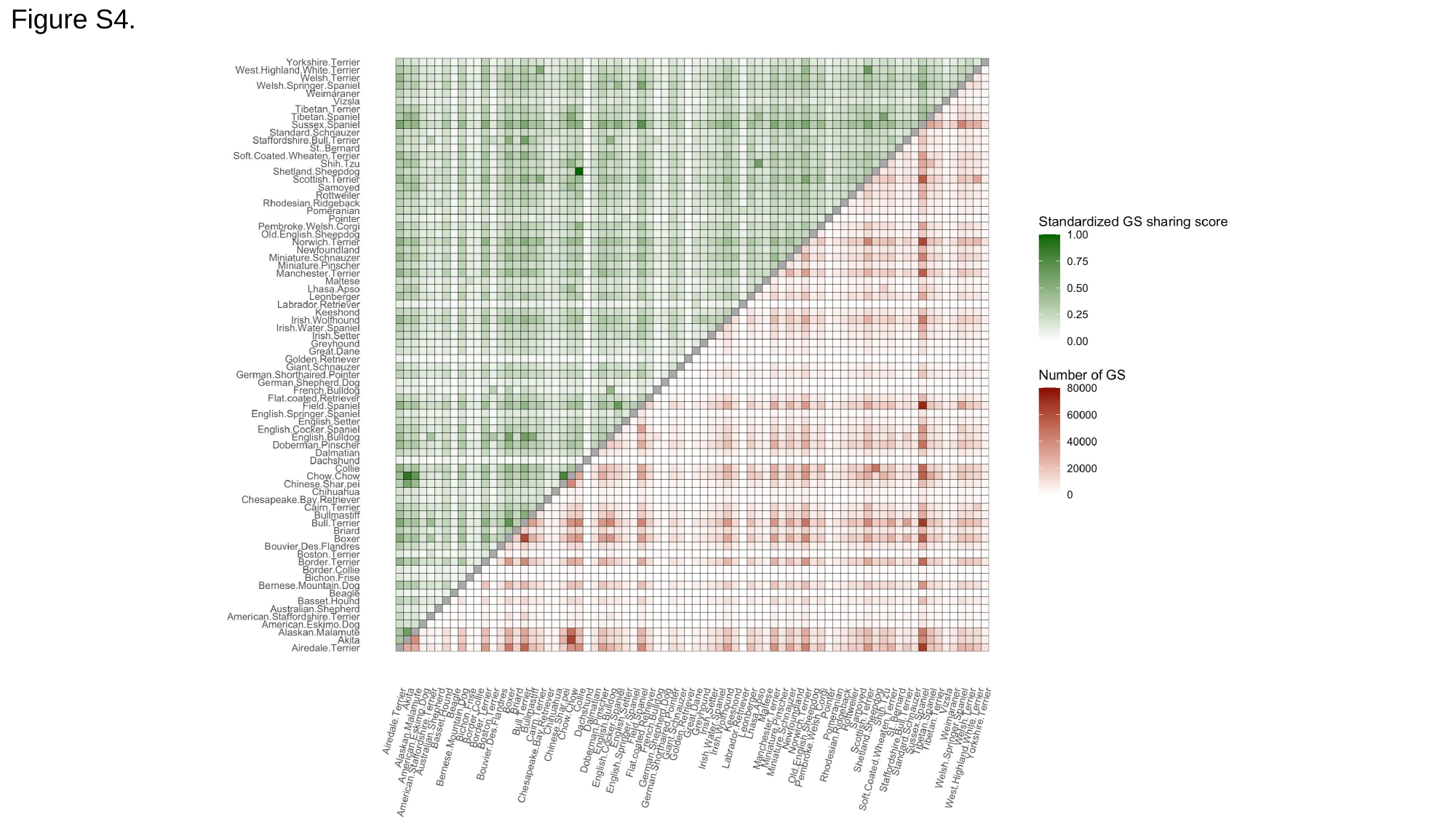

Figure S4.

## Slide 6
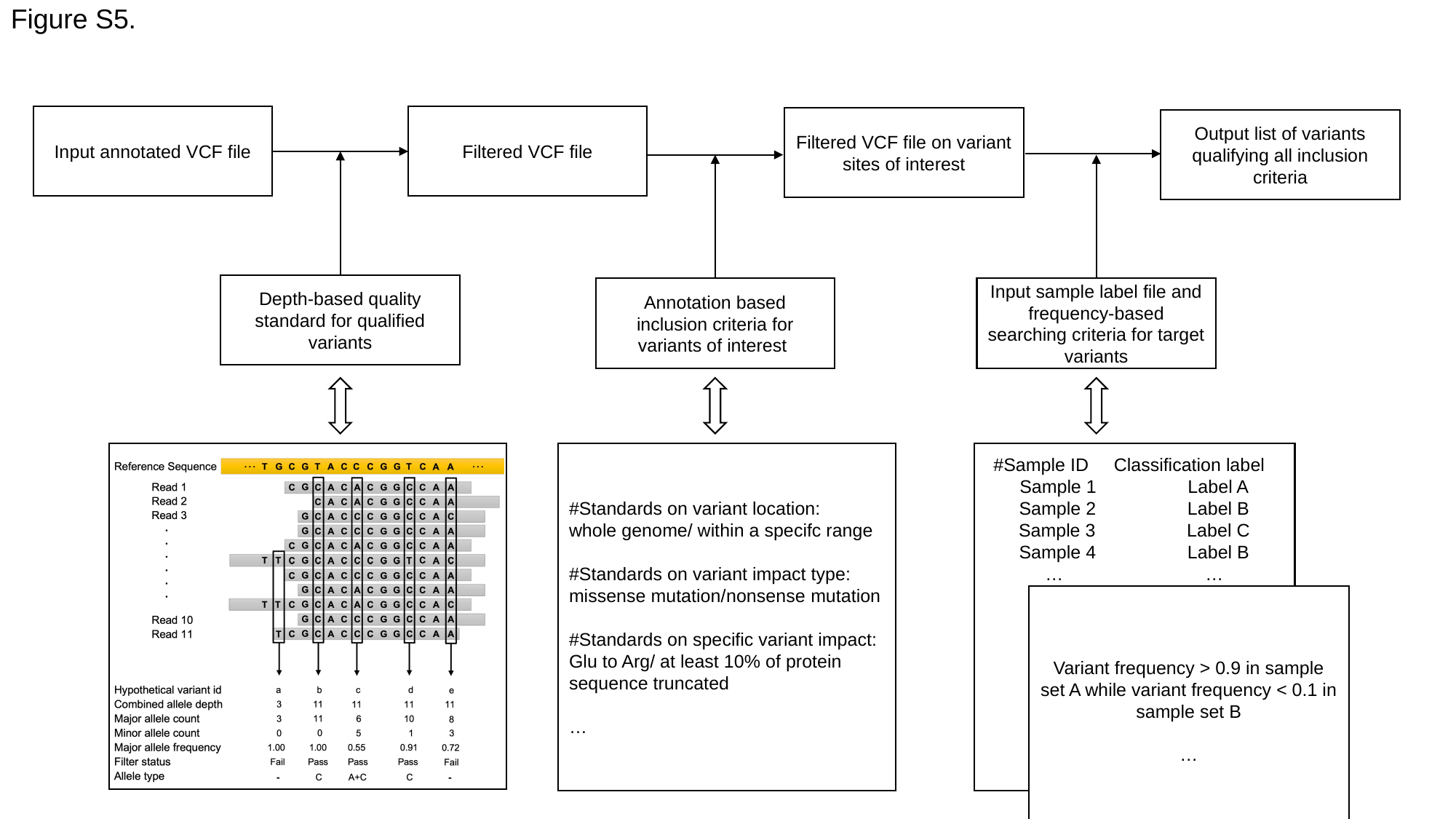

Figure S5.
Input annotated VCF file
Filtered VCF file
Filtered VCF file on variant sites of interest
Output list of variants qualifying all inclusion criteria
Depth-based quality standard for qualified variants
Annotation based inclusion criteria for variants of interest
Input sample label file and frequency-based searching criteria for target variants
…
#Standards on variant location:
whole genome/ within a specifc range
#Standards on variant impact type:
missense mutation/nonsense mutation
#Standards on specific variant impact:
Glu to Arg/ at least 10% of protein sequence truncated
…
#Sample ID Classification label Sample 1 Label A
Sample 2 Label B
Sample 3 Label C
Sample 4 Label B
… …
Variant frequency > 0.9 in sample set A while variant frequency < 0.1 in sample set B
…

## Slide 7
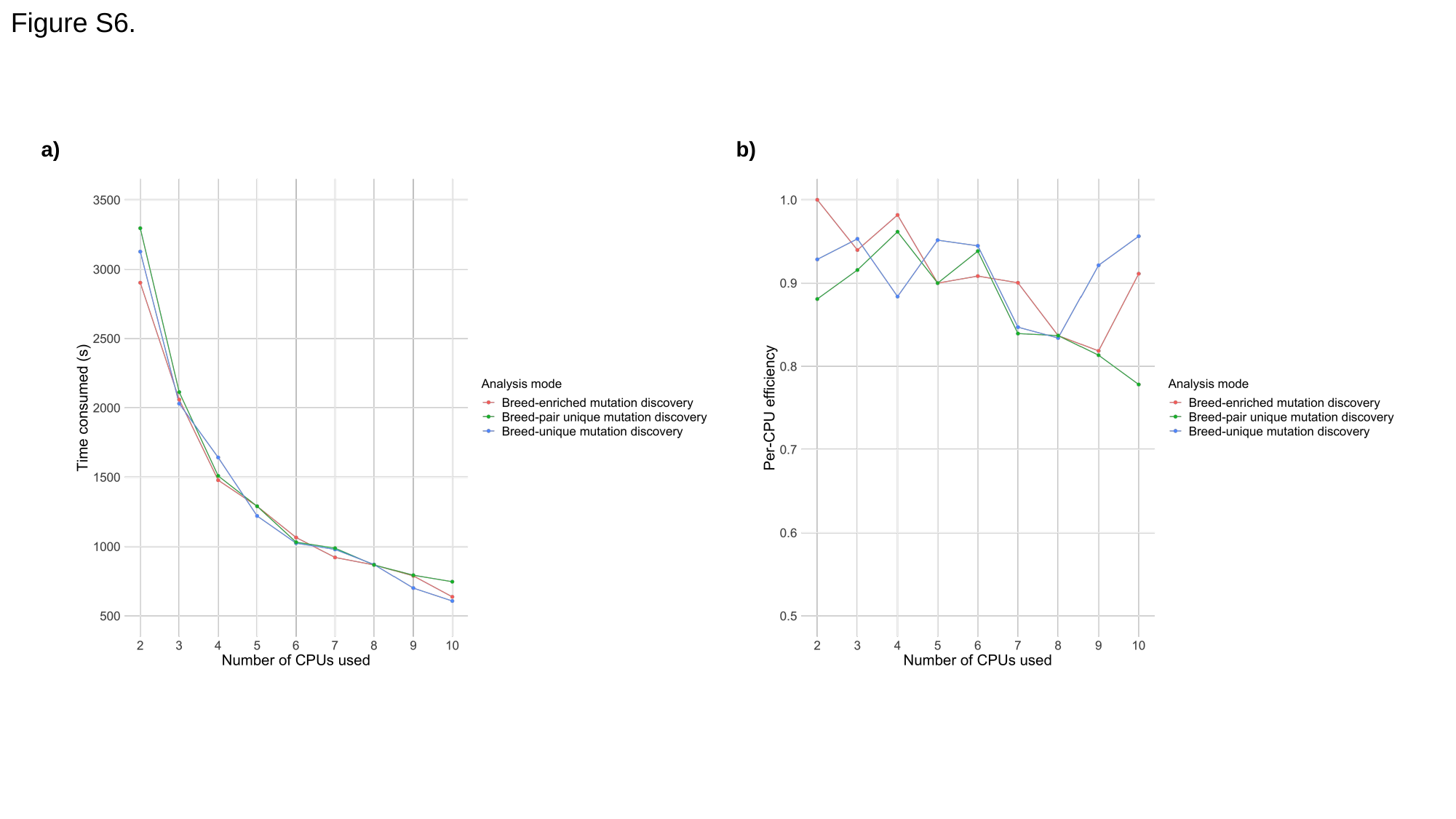

Figure S6.
a)
b)
